# Spectrotemporal cues and attention jointly modulate fMRI network topology for sentence and melody perception

**DOI:** 10.1038/s41598-024-56139-6

**Published:** 2024-03-06

**Authors:** Felix Haiduk, Robert J. Zatorre, Lucas Benjamin, Benjamin Morillon, Philippe Albouy

**Affiliations:** 1 Department of Behavioral and Cognitive Biology, University of Vienna, Vienna, Austria; 2 Department of General Psychology, University of Padua, Padua, Italy; 3 Cognitive Neuroscience Unit, Montreal Neurological Institute, McGill University, Montreal, QC Canada; 4 International Laboratory for Brain, Music and Sound Research (BRAMS) - CRBLM, Montreal, QC Canada; 5 Cognitive Neuroimaging Unit, CNRS ERL 9003, INSERM U992, CEA, Université Paris-Saclay, NeuroSpin Center, 91191 Gif/Yvette, France; 6 Aix Marseille University, Inserm, INS, Institut de Neurosciences des Systèmes, Marseille, France; 7 CERVO Brain Research Centre, School of Psychology, Laval University, Quebec, QC Canada

**Keywords:** Attention, Language, Perception

## Abstract

Speech and music are two fundamental modes of human communication. Lateralisation of key processes underlying their perception has been related both to the distinct sensitivity to low-level spectrotemporal acoustic features and to top-down attention. However, the interplay between bottom-up and top-down processes needs to be clarified. In the present study, we investigated the contribution of acoustics and attention to melodies or sentences to lateralisation in fMRI functional network topology. We used sung speech stimuli selectively filtered in temporal or spectral modulation domains with crossed and balanced verbal and melodic content. Perception of speech decreased with degradation of temporal information, whereas perception of melodies decreased with spectral degradation. Applying graph theoretical metrics on fMRI connectivity matrices, we found that local clustering, reflecting functional specialisation, linearly increased when spectral or temporal cues crucial for the task goal were incrementally degraded. These effects occurred in a bilateral fronto-temporo-parietal network for processing temporally degraded sentences and in right auditory regions for processing spectrally degraded melodies. In contrast, global topology remained stable across conditions. These findings suggest that lateralisation for speech and music partially depends on an interplay of acoustic cues and task goals under increased attentional demands.

## Introduction

Spoken language and singing are two fundamental modes of human communication. As vocalised instances of language and music, respectively, they share several structural features^1–6^. However, song and speech also differ in numerous ways. Notably, the formation of hierarchical tonal organisation^7^ in music is typically related to discrete pitches arranged in scales (forming melodies) rather than continuous pitch (gliding intonation), such as typical in speech^1,8^).

Recent studies have offered a framework to explain such differences between speech and song by showing that complex sounds can be characterised by the distribution of their spectrotemporal modulation (STM) power^9^. Over the last decades, it has been demonstrated that (i) syllable rate of speech is associated with a high rate of temporal modulation^10^ resulting in faster temporal modulations as compared to music^10,11^, whereas syllable duration in singing is longer, and enables the production of stable pitch values for a better encoding of tonal relationships^12^; (ii) the complex physiology of phonation typical of singing (i.e. energy in the upper harmonics) is associated with higher spectral modulation rate^13,14^ than speaking; (iii) even when spectral modulations are degraded, temporal modulation cues are sufficient for perceiving speech^15^; (iv) degradation of spectral modulations considerably reduces the perception of melodic content in song, but does not affect speech comprehension, while (v) degradation of temporal modulations reduces speech comprehension but has little or no effect on melody perception^16^, and (vi) spectrotemporal modulation differences between song and speech are found across several cultures^17^.

Recent studies have suggested that song and speech are preferentially processed in distinct auditory cortical regions because of hemispheric specialisation in sensitivity to spectral and temporal modulations^16^: the right auditory cortex has higher spectral resolution but lower temporal resolution whereas the left auditory cortex has the reverse specialisation^18–21^. According to this framework, music and speech utilise opposite ends of the spectrotemporal continuum^16^, explaining their hemispheric lateralisation. However, speech and music also appear to occupy distinct cortical regions beyond mere acoustics processing^22,23^, or at least distinct spectral fingerprints, which consist of network- and frequency-specific coherent neural dynamics^24^. Indeed, despite spectrotemporal differences being processed by distinct brain regions, top-down factors likely influence the perception of a vocalisation as song or as speech, as illustrated by the “speech-to-song” illusion wherein acoustics are held constant^25^. Left-hemispheric lateralisation has, for example, been observed when listeners focus to propositionally meaningful features of spoken language (e.g. prosodic intonation, Ref.^26^). Even in the absence of any acoustic input, speech imagery experiments have shown left-lateralised activations, depending on emotional valence^27,28^. Moreover, acoustically identical stimuli perceived as song rather than speech appear to preferentially recruit the right temporal cortex^29^. Auditory attention and prediction can interact in different ways depending on task demands (or work independently, see e.g. Ref.^30^). For example, when attending one of two sound streams based on temporal cues, temporal predictions originate in the left sensorimotor cortex^31^. Therefore, top-down attention can be expected to interact with acoustic cues and influence the spectrotemporal hemispheric lateralisation in speech and song perception^32^.

In the present study, we asked whether brain asymmetry for speech and music is modulated by both task goals (mediated by top-down attention) and spectrotemporal acoustic information provided in the stimulus, or by acoustical cues only. We considered two alternative hypotheses: (1) explicit attentional focus to melodies or sentences of sung speech interacts with spectral or temporal acoustic cues to contribute to hemispheric lateralisation (interaction hypothesis), or (2) hemispheric lateralisation depends solely on bottom-up acoustic information, that is spectral or temporal acoustic cues (acoustic hypothesis). Both theoretical work and empirical results have shown that cognitive processing, including attention, is dynamic and can be both localised and distributed, which makes the investigation of functional connectivity highly valuable^33–36^. The way in which bottom-up spectrotemporal and top-down attentional processing interact dynamically as a function of task-goals to influence lateralisation remains to be investigated^37^. To our knowledge, the current study is the first one investigating cortical functional connectivity of attention in the framework of spectrotemporal modulation for speech and song.

We, therefore, manipulated both the spectral or temporal acoustic information available in sung speech stimuli and the task goal, which was to recognise either the sentence or the melody (for simplicity, we will refer to the sentence aspect as "sentence" or "speech" in this paper, despite lacking prosodic features^38^). Using behavioural and fMRI data from Ref.^16^, we examined the corresponding modulation of brain connectivity patterns manifested in network topology of functional connectivity networks.

Since attention-related effects can be expected both in distributed networks^39^ and single hub regions that are integrated with surrounding networks (e.g. Ref.^40^), we used graph theoretical metrics^41,42^. Graph theory investigates the topology of a network, that is, the way single regions ("nodes") are connected, revealing how networks subserve functional dynamics (e.g. integration or segregation). It can characterise several topological properties at global (the entire network), medium (subnetworks) and local (hub regions) levels of networks and is therefore well suited for functional connectivity analyses.

Previous work showed that topological network properties are modulated dynamically to cope with different listening situations^43,44^. For spatial attention to two competing speech streams, Alavash and colleagues observed a dynamical reconfiguration of brain network connectivity, altering functional integration and segregation relative to resting-state, in auditory, ventral attention network, and cingulo-opercular regions^43^. Integration and segregation were measured by different graph metrics such as "clustering coefficient" (i.e. the proportion of neighbours of a node that are also connected to each other), "efficiency" (i.e. the inverse of the shortest path between two nodes in the graph), and the network modularity (i.e. the organization of nodes in subnetworks forming quasi-independent clusters). Specifically, increased modular segregation (i.e. more localised processing) within the auditory fronto-temporal control network predicted individuals' listening success in this study. Along with modular segregation^43^, reported an increase in local but a decrease in global efficiency with more challenging speech comprehension relative to resting-state. "Local" here refers to the metrics for a specific node, while "global" refers to the average metric across all nodes in the network. Thus, these measures are related to localised (subnetwork) or global (entire network) integration of information, respectively^42^. Similarly, Ref.^44^ observed increased clustering and efficiency in auditory modules when attending to auditory (piano-like chords) instead of visual stimuli in an auditory-visual task. Finally, Ref.^43^ suggested that functional segregation was a general principle of auditory attention. Thus, if attention was directed either to sentences or to melodies in sung speech stimuli, such segregation might be lateralised between song and speech.

We, therefore, predicted the following for the present study: If lateralisation for speech vs music depended on the interaction of task goal (attention to melody or sentence) and affordances (acoustic cues) we would expect to find a hemispheric difference in modular segregation, scaling under attentional demands (interaction hypothesis). We also predicted to observe increased local clustering and local efficiency but decreased global efficiency when task-relevant acoustical cues are degraded. We expected these effects to be lateralised (left for attention to sentences and temporal degradation, right for attention to melodies and spectral degradation) in auditory, cingulo-opercular and/or ventral attention networks. On the contrary, if lateralisation for speech vs music depended only on acoustical cues (acoustic hypothesis), we would expect lateralisation to occur independent of task goals (mediated by attention), solely based on acoustical cues.

## Results

**Figure 1:**
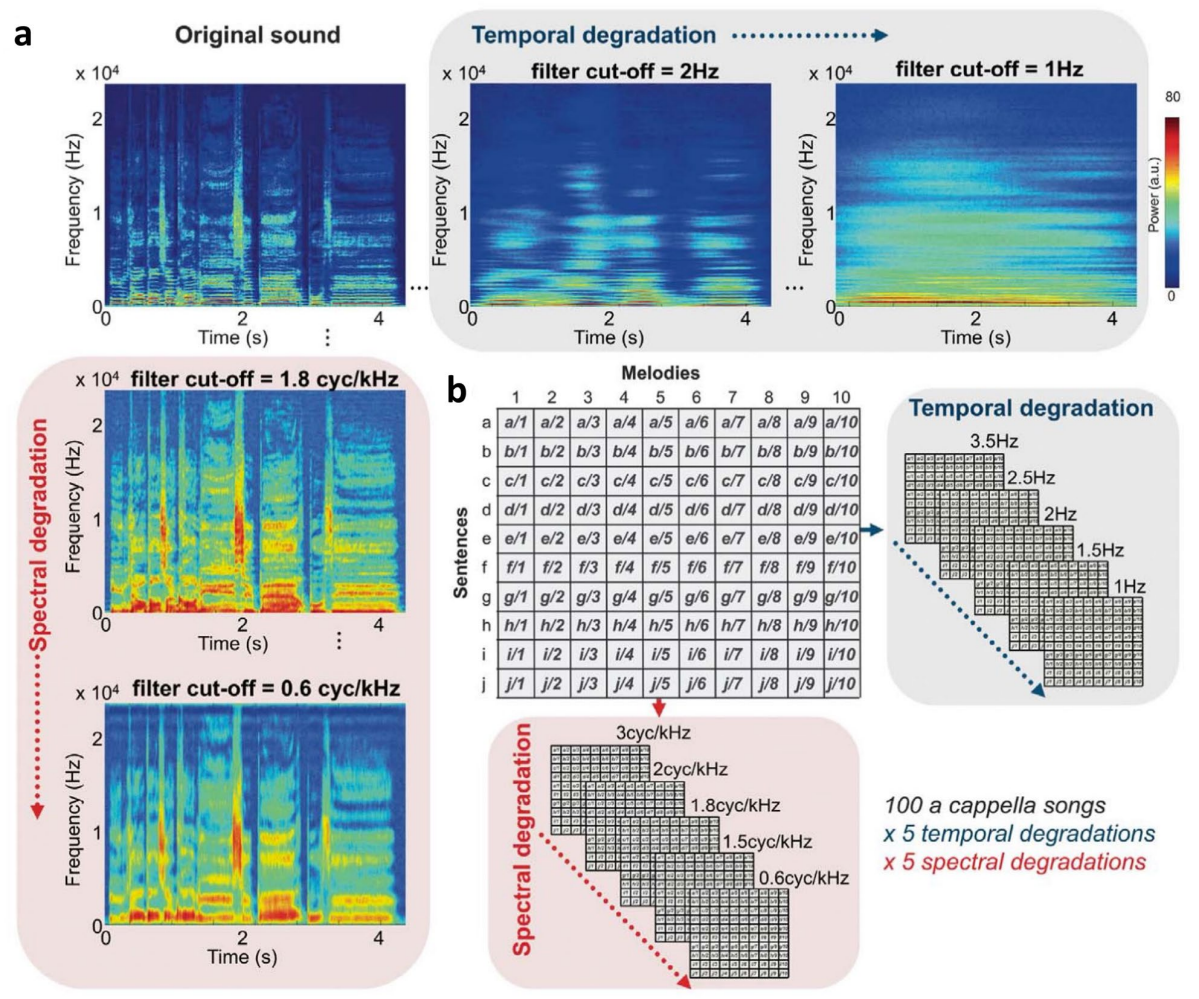
Spectrotemporal filtering and stimulus set. (**a**) Spectral and temporal degradations applied on an original a cappella song. (**b**) One hundred a cappella songs in each language were recorded following a 10 × 10 matrix with ten melodies (number code) and ten sentences (letter code). Stimuli were then filtered either in the spectral or in the temporal dimension, with 5 filter cut-offs, resulting in 1000 degraded stimuli for each language (taken with permission from Ref.^16^).

The behavioural and fMRI data used in the present study are a subset of the data presented in Ref.^16^ (i.e., the 15 out of 27 French-speaking individuals who performed both the behavioural task and the fMRI session). In this previous study, the authors created a unique stimulus set in which ten original sentences were crossed with ten original melodies, resulting in 100 naturalistic acappella songs (Fig. 1, stimuli available at: https://osf.io/9ub78/). This orthogonalisation of speech and melodic domains across stimuli allows for the dissociation of speech- (or melody-)specific acoustic features from non-specific acoustic features, thereby controlling for any potential acoustic bias^45^. The authors created two separate stimulus sets, with French and English sentences. In the present study, we only used data recorded with the French set. The authors then parametrically degraded each stimulus selectively in either the temporal or spectral dimension using a manipulation that decomposes the acoustical signal using the Spectrotemporal Manipulation framework^46^.

### Behavioural results

**Figure 2:**
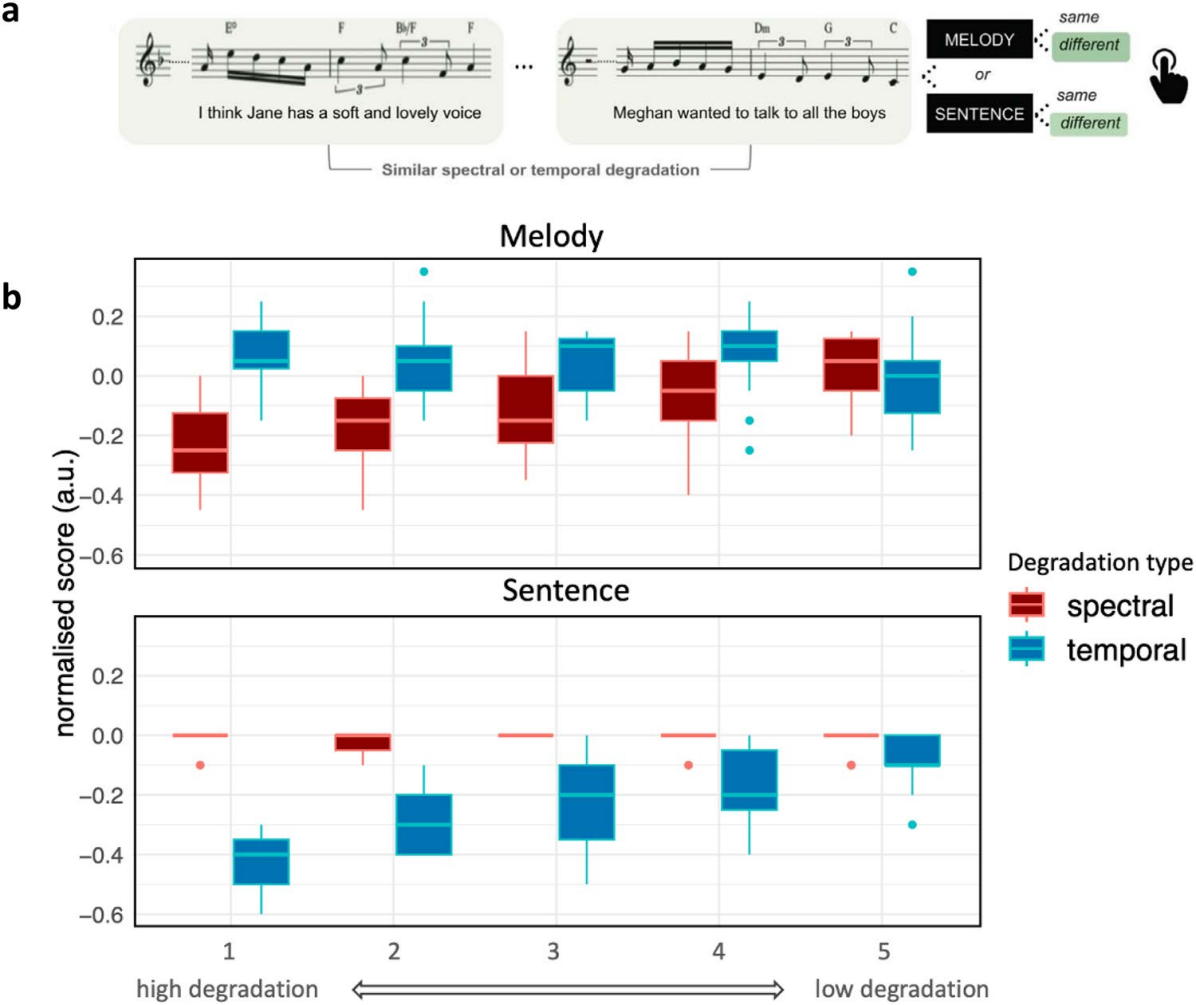
Behavioural task. (**a**) 15 French-speaking participants listened to degraded (either in the spectral or temporal dimension) a cappella songs presented in pairs. After the second song, a visual instruction indicated the domain of interest (sentences or melodies). The lower panel shows example trials (adapted from Ref.^16^ with permissions). (**b**) Behavioural performance of 15 French-speaking listeners who participated in both behavioural and fMRI experiments presented as a function of task instructions (judging melodies or sentences) and degradation cut-off values. Blue bars: Temporal degradations; Red bars: Spectral degradations. Boxplot whiskers indicate first quartile − 1.5 * interquartile range and third quartile + 1.5 * interquartile range, respectively. The nonsignificant variability between degradation steps for melody recognition and temporal degradation is likely due to little musical training in our participants.

We first aimed to confirm the importance of spectrotemporal rates on sentence or melody recognition scores (Fig. 2A) in the subset of native French-speaking (n = 15) listeners from Ref.^16^ (who performed both the behavioral and fMRI experiment). Participants were presented with pairs of stimuli and asked to discriminate either the speech or the melodic content. Thus, the stimulus set across the two tasks was identical, only the instructions differed. To manipulate the amount of spectral or temporal acoustic information, stimuli were degraded in the modulation power spectrum domain either temporally or spectrally with 5 cut-off values each (see Fig. 1 and “Materials and methods”), leading to different amounts of acoustical information retained. For each participant, the modulation of performance between degraded and original (baseline, non-degraded) stimuli was computed as follows:

$$\mathrm{normalized\, score}= \frac{\left(\mathrm{raw \,score}-\mathrm{ chance}\right)}{\left(\mathrm{baseline }-\mathrm{ chance}\right)}-1$$

with chance corresponding to 50% correct performance. The baseline score corresponds to performance obtained with the original songs (without degradations) for each participant. A normalised score of 0 indicates no decrease in performance as compared to baseline, while positive and negative scores, respectively, indicate beneficial and deleterious effects on accuracy. We chose this strategy to consider individual differences in overall melody or sentence recognition (without degradation). Behavioural results were computed based on these normalised deviation scores from the baseline, integrated over trials using a linear function for each participant (see “Materials and methods”).

We fitted a linear model with the interaction of "cutoff" (1 to 5), "task" (sentences or melodies), and "degradation" (temporal or spectral) as predictors. Comparing the full model with a reduced model lacking the predictors of interest revealed a significant interaction (F_13, 280_ = 22.154, p < 0.001, R^2^_adj_ = 0.555) with behavioural performance decreasing as a function of degradation, task, and cutoff (see Fig. 2B and Supplementary Material for detailed statistics). More precisely, the degradation of information in the temporal dimension impaired sentence recognition but not melody recognition, while the degradation of information in the spectral dimension impaired melody recognition but not sentence recognition. No collinearity issues were found.

The number of 15 participants was enough to detect an effect for the behavioural data, as we confirmed with a power analysis based on the full dataset of Ref.^16^ (R^2^ = 0.1533, df = 19, res.df = 1010). The power analysis revealed sufficient power of 0.99 for a significance level of 0.05 with the parameters above for 300 observations (≙ 15 participants). Note that post-hoc power analyses are generally not recommended (e.g. Refs.^47,48^; see also Ref.^49^). A sample size of 15 is comparable to other fMRI studies in the field (e.g. N = 10 in Ref.^22^; N = 20 in Ref.^50^; N = 13 in Ref.^51^).

### fMRI results

**Figure 3:**
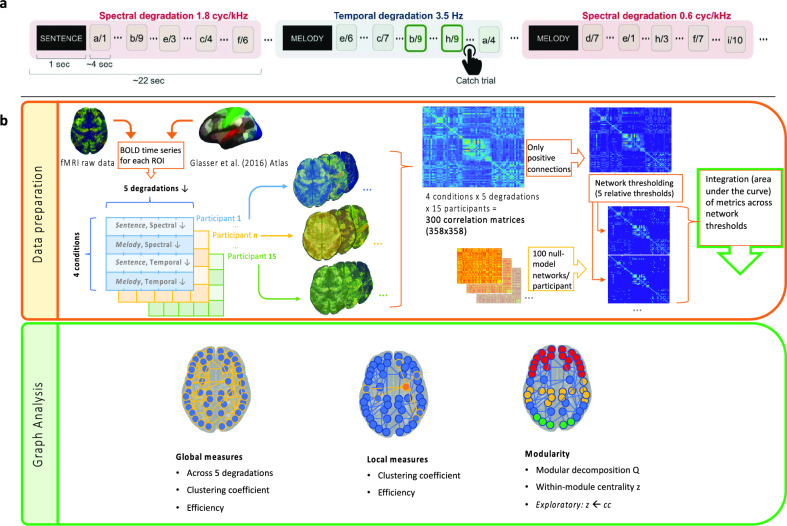
fMRI task and analysis. (**a**) fMRI design: BOLD activity was collected while participants listened to blocks of 5 sung-speech stimuli (degraded in the temporal or spectral dimension). To modulate attention, participants were asked to detect two catch trials (with the high filter cut-off (low degradation): 3cyc/kHz, and 3.5 Hz) containing melody (or sentence) repetition (1-back task) (taken from Ref.^16^ with permission). (**b**) Overview of data preprocessing to enter the graph theoretical analyses (upper panel) and the different graph theoretical analyses (lower panel). We created ROI-to-ROI correlation matrices (Fisher z-transformed) as a function of attention (sentences/melodies), degradation type (spectral/temporal) and cutoff value (5 steps per degradation type) for each of the 15 participants. This resulted in 300 358 × 358 functional connectivity matrices (20 per participant, see Table 1). We then applied several analyses using measures from Graph Theory, a field that investigates networks regarding their topological organisation. It models brain connectivity networks as "nodes" (ROI) connected by "links" (or "edges"), which in our case were the Fisher z-transformed correlations across ROIs. Graph measures can be calculated regarding the entire functional connectivity network (global measures) and subnetworks or single nodes (local measures).

Blood-oxygenation level-dependent (BOLD) activity was recorded while the 15 French speakers who had participated in the behavioural experiment listened to blocks of five songs degraded either in the temporal or spectral dimension. Participants were asked to attend either to the melody or the sentence of a stimulus, visually cued before each of 110 blocks of five degraded stimuli, resulting in a 2 (attention) × 2 (degradation type) × 5 (degradation strength) design. The participants’ task was to detect repetitions of sentences or melodies (1-back task, see Fig. 3A). We will abbreviate the four attention x degradation conditions as follows throughout the paper: MS: attention to melody, spectral degradation (temporal acoustic cues retained), MT: attention to melody, temporal degradation (spectral acoustic cues retained), SS: attention to sentences, spectral degradation (temporal acoustic cues retained), ST: attention to sentences, temporal degradation (spectral acoustic cues retained).

**Table 1:** Likelihood-ratio tests for ROI with significant full-null model comparisons for the local clustering coefficient.

ROI	Effect	Sum Sq	Mean Sq	NumDF	DenDF	F	Pr(> F)
l8BM	**Attention:degradation:cutoff**	**0.003**	**0.001**	**4.000**	**263.361**	**3.753**	**0.005**
lH	Attention:degradation:cutoff	0.003	0.001	4.000	203.825	1.996	0.096
	**Cutoff:attention**	**0.004**	**0.001**	**4.000**	**226.328**	**2.564**	**0.039**
	**Cutoff:degradation**	**0.007**	**0.002**	**4.000**	**226.328**	**4.808**	**0.001**
	Attention:degradation	0.000	0.000	1.000	226.328	1.012	0.316
rA5	**Attention:degradation:cutoff**	**0.005**	**0.001**	**4.000**	**239.211**	**6.075**	**0.000**
rAIP	**Attention:degradation:cutoff**	**0.004**	**0.001**	**4.000**	**224.421**	**3.196**	**0.014**
rSTSvp	**Attention:degradation:cutoff**	**0.003**	**0.001**	**4.000**	**240.218**	**3.620**	**0.007**

Highest order effects that are significant are in bold.

In order to investigate how connectivity patterns changed depending on spectrotemporal acoustic cues and attention to speech or melody, we calculated complementary graph theoretical measures using the GraphVar toolbox^52,53^ in Matlab. This allowed us to measure the entire network's behaviour, the differential emergence of subnetworks, and the functional role of single regions of interest (ROI) in a statistically rigorous way. We used a parcellation by Glasser et al.^54^ resulting in a network of 358 ROI. We thresholded the 358 × 358 connectivity matrices (see Fig. 3 and “Materials and methods”, consisting of Fisher z scores derived from correlation coefficients between ROI) for each subject to exclude spurious or noisy weights by using several thresholds to prevent measures from being biased (see “Materials and methods”). We conducted several confirmatory analyses based on Graph measures (see below) and an exploratory analysis (network-based statistic, see Supplementary Material).

### Results of graph measures

In the present study, we asked whether brain asymmetry for speech and music is modulated by both available spectral or temporal acoustic information and task goals (interaction hypothesis) or by acoustical cues only (acoustic hypothesis). Based on previous findings on attentional effects on functional connectivity in auditory processing, we derived graph theoretical measures at global and local levels of connectivity as well as network modularity. We expected this approach to clarify how localised processes interacted with wide ranging attentional networks in lateralisation for song and speech. Thus, based on the literature, we predicted effects for specific graph measures: efficiency, clustering, and modularity.

We calculated the clustering coefficient and the efficiency both for the entire network for each ROI (local) and the average across all ROIs to obtain global measures for the entire network (global). Since we applied multiple thresholds to the connectivity matrices, we used the area under the curve across all thresholds to obtain an integrated measure of global and local clustering coefficient and efficiency, respectively^42,55,56^. For simplification, we will further refer to these integrated measures as local and global clustering coefficient and efficiency, respectively. We also analysed network modularity as a further global metric.

We used Generalised Linear Mixed Models (GLMM), including the fixed factors "attention" (speech or melody), "degradation" (spectral or temporal degradation), "cutoff" (5 cutoffs from high to low degradation) and their interactions. We also included participants as random effects and random slopes of all fixed effects within participants. All statistical analyses were done in R^57^.

### Global graph measures

#### Global clustering coefficient

The clustering coefficient measures what fraction of the neighbours of a given node in a network are also connected among each other, forming closed triangular patterns (motifs). In our case, neighbours are ROI with connectivity retained after thresholding the entire connectivity matrices. The global clustering coefficient measures the mean clustering coefficient across all ROI. The clustering coefficient can be interpreted as integration within triangular node motifs, which could support functional specialisation (it does, however, not directly measure segregation; see Ref.^42^).

We calculated Variance Inflation Factors^58^ to assess collinearity between the three fixed predictors of our GLMM but did not observe any collinearity issues ﻿(R package “car”; version 3.0–2; Ref.^59^). Model stability was assessed by comparing the full model estimates with estimates obtained from reduced models with levels of the random effects excluded one at a time. The model was stable regarding fixed effects estimates (regarding random effects estimates were not as stable; however, random effects were not the focus of these models). We obtained 0.95 confidence intervals using the function “bootMer” (package “lme4”, version 1.1–21; Ref.^60^ with 1000 parametric bootstraps (see Supplementary Material).

The comparison of the full model with a null model devoid of fixed effects terms was significant (χ^2^ = 41.358, df = 19, p = 0.002, R^2^m = 0.01, R^2^c = 0.297). Thus, the predictors of interest explained the data significantly better than the null model.

We then tested the effects of the predictors using likelihood-ratio tests, comparing the full model with models reduced by the predictors of interest (R function “drop1” with argument “test” set to “Chisq”). The three-way interaction "attention:degradation:cutoff" was significant (LRT = 13.124, AIC = -722.523, p = 0.01, R^2^m = 0.01, R^2^c = 0.297). Thus, lower-order effects (two-way interactions and main effects) were conditional on each other and not meaningful to interpret independently.

To test which cutoffs drove the interaction "attention:degradation", we fitted GLMMs for each cutoff separately and compared the resulting full (fixed effects "attention" by "degradation", random effect "participant", random slopes of fixed effects within "participant") and null models as post-hoc tests.

However, we did not obtain a clear pattern across cutoffs (see Supplementary Fig. S1).

#### Global efficiency

Global efficiency is the inverse of the average shortest path length of the network. Both path length/efficiency and clustering are two fundamental network characteristics^41^. Efficiency can be interpreted as measuring communication facility between nodes and therefore as a measure of integration. We used the same model structure and diagnostic functions as for the global clustering coefficient. Model stability was acceptable in terms of fixed effects (regarding random effects estimates were not as stable, however, random effects were not the focus of these models). We did not obtain any collinearity issues. The full model explained the data significantly better than the null model (χ^2^ = 39.173, df = 19, p = 0.004, R^2^m = 0.109, R^2^c = 0.231). As for the global clustering coefficient, the three-way interaction "attention:degradation:cutoff" was significant ﻿(LRT = 17.514, AIC =  − 2121.311, p = 0.002, R^2^m = 0.109, R^2^c = 0.231). As for the global clustering coefficient we fitted separate models with the "attention:degradation" interaction as fixed effects (random effect "participant", random slopes of fixed effects within "participant") for each cutoff and ran full-null model comparisons as post-hoc tests. However, we did not obtain a clear pattern across cutoffs (see Supplementary Fig. S2).

#### Modularity

We investigated whether segregation in network modularity increased as a function of attention to melody or sentence and spectral or temporal degradation across cutoffs. Using GraphVar, we ran 1000 iterations of the Louvain algorithm for each condition, cutoff and participant, with a resolution factor gamma of 1.0 (standard value), to derive the subject-level community structure and the metrics Q. The modularity index Q designates the optimal modular decomposition of the network, which means that nodes within a module are more connected to each other than to nodes outside the module. A higher Q value therefore indicates more functional segregation (see Ref.^43^).

We used the Q values as response variable in a GLMM with the fixed factors "attention", "degradation", and "cutoff". We included the three-way interaction of these factors, as well as "participant" as random factor and random slopes of all fixed factors within "participant" (i.e., the same model structure as for clustering coefficient and efficiency). The model diagnostics revealed no collinearity issues and acceptable model stability regarding fixed effects (the model was not very stable regarding random effects; however, random effects were not the focus of this model). The full-null model comparison remained not significant (χ^2^ = 15.632, df = 19, p = 0.682). Thus, although some ROI changed their affiliation with given modules by condition (see OSF repository https://osf.io/merwk/), the results indicate no significant difference in the overall modular segregation across conditions and cutoffs. We additionally analysed whether any of the ROI would change in their within-module centrality, however we did not observe any significant changes (see Supplementary Material).

### Local graph measures

#### Local clustering coefficient

**Figure 4:**
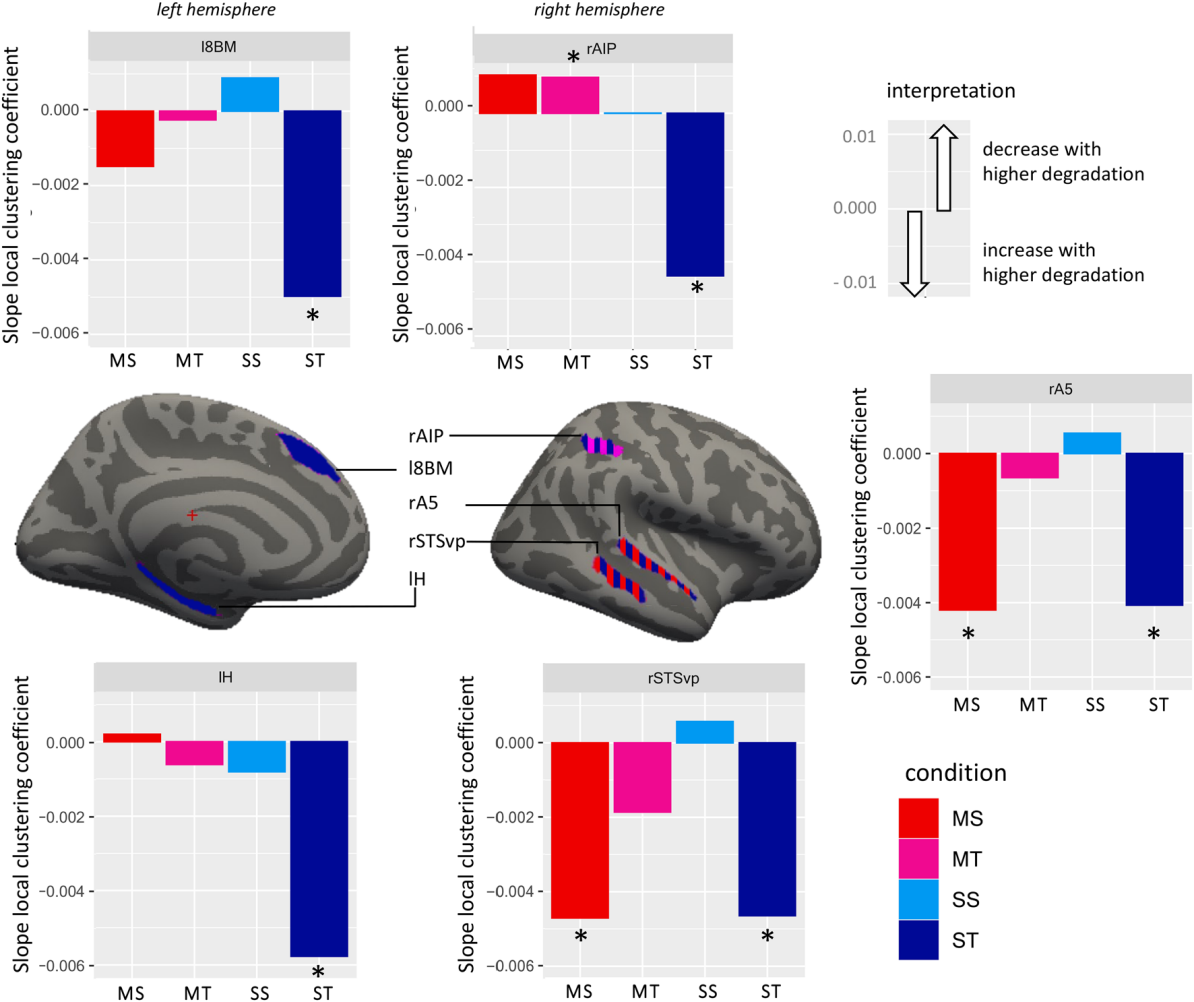
ROI with significant full-null model comparisons for the local clustering coefficient, and their respective model slope values (see Table 3) for each condition. Negative slopes indicate a decrease of local clustering with lower degradation intensity (cutoff), or likewise an increase with higher degradation intensity. Significant effects for degradation intensity occurred for ST bilaterally, thus in all the five ROI of this subnetwork, and for MS for right STSvp and right A5 (see also Fig. 5). Asterisks indicate significance. Brain regions are colored according to significant effects. Brain images throughout the paper have been done with FreeView (version 3.0) of the FreeSurfer package^61^.

Next, we calculated the clustering coefficient for each ROI, indicating the integration of each ROI with its neighbourhood in closed-triangle motifs. We used the same model structure as for the global clustering coefficient, fitting a model for each of the 358 ROI. After FDR correction, full-null model comparisons of models for 5 ROI were significant (see Fig. 4, Supplementary Table S4). These ROI form a bilateral network comprising left medial prefrontal (l8BM), left hippocampal (lH), and right auditory associative regions (rA5, rSTSvp), and right superior parietal (rAIP) region. We then conducted likelihood ratio tests for the three- and two-way interactions (see Table 1).

**Figure 5:**
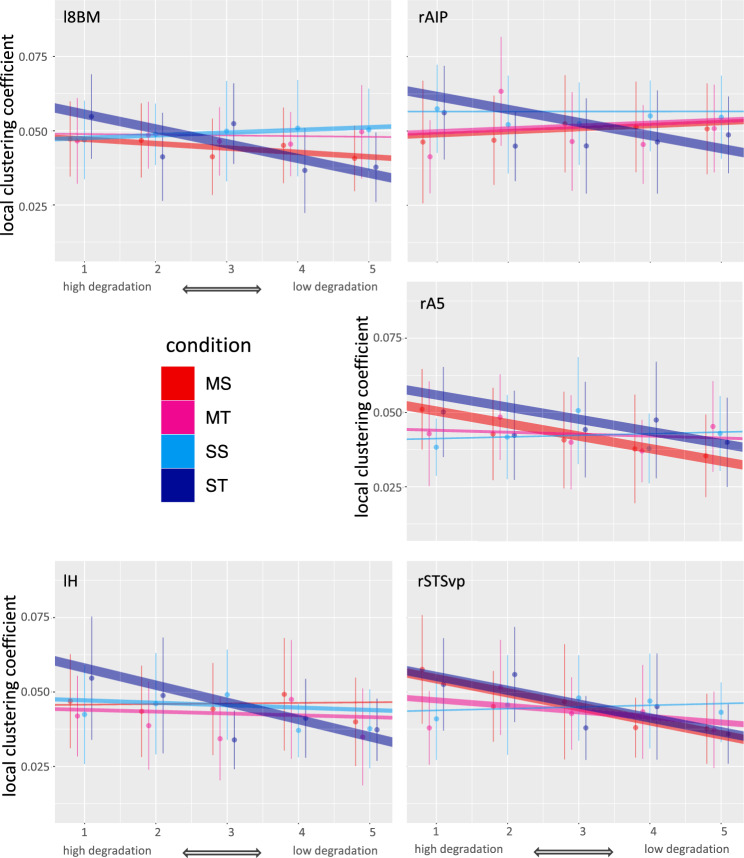
Mean, SD and model slopes for each significant region for the local clustering coefficient network, across cutoffs (high to low degradation). More extreme slope values are depicted with higher line thickness.

To see whether for any of the conditions, there was an effect of degradation intensity (cutoff) at any of the five ROI, we conducted post-hoc full-null model comparisons for each ROI and condition separately, with "cutoff" as fixed and "participant" as random effect in the full model. FDR-corrected results revealed that for ST (attention to sentences with temporal degradation) in all five ROI, thus bilaterally, the local clustering coefficient increased significantly with higher degradation, while for MS (attention to melodies with spectral degradation) this increase occurred in the right-hemispheric ROI rA5 and rSTSvp. At rAIP and for MT (attention to melodies with temporal degradation), local clustering decreased across cutoffs (see Table 2 and Figs. 4 and 5).

**Table 2:** Results for post-hoc full-null model comparisons regarding the effect of degradation intensity (cutoff) for the five ROIs of the local clustering coefficient subnetwork.

ROI	Condition	df	Intercept	Slope	χ^2^	p _(FDR corrected)_	R^2^m	R^2^c
l8BM	ST	4	0.061	− 4.97E − 03	25.938	0.001	0.275	0.325
lH	ST	4	0.064	− 5.77E − 03	20.595	0.003	0.223	0.280
rA5	MS	4	0.055	− 4.20E − 03	13.110	0.031	0.136	0.265
rA5	ST	4	0.060	− 4.07E − 03	12.337	0.038	0.112	0.354
rAIP	MT	4	0.049	9.65E − 04	17.196	0.007	0.162	0.356
rAIP	ST	4	0.066	− 4.38E − 03	16.623	0.008	0.171	0.293
rSTSvp	MS	4	0.059	− 4.70E − 03	17.540	0.007	0.197	0.228
rSTSvp	ST	4	0.060	− 4.64E − 03	22.670	0.001	0.250	0.250

We investigated whether ROI could be specifically associated with sentence, melody, temporal or spectral processing by conducting post-hoc likelihood-ratio tests at each cutoff but did not obtain clear results regarding our hypotheses (see Supplementary Material).

#### Local efficiency

**Figure 6:**
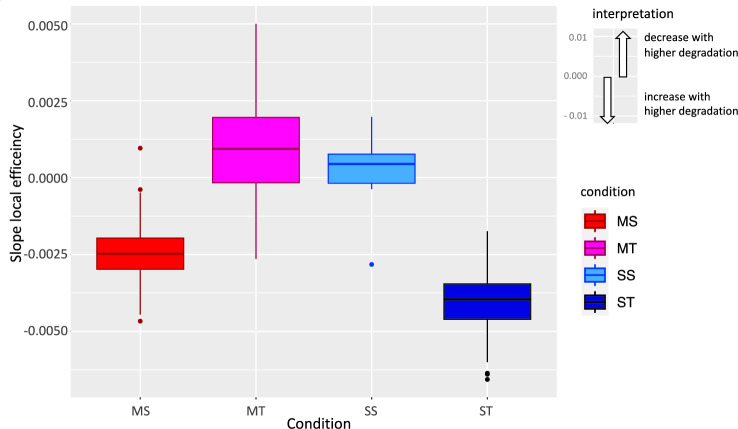
Model slope values across significant ROI for local efficiency. Negative values indicate a decrease of efficiency with lower degradation intensity (cutoff) or, equivalently, an increase of efficiency with higher degradation intensity, as can be obtained clearly for MS and ST. Of all ROI with significant changes in local efficiency across cutoffs, most showed effects for MS (107 ROI increasing with higher degradation, 1 decreasing with higher degradation) and ST (213, 0). In contrast, only few ROI showed significant effects for SS (2, 4) and MT (3, 8). Boxplot whiskers indicate first quartile − 1.5 * interquartile range and third quartile + 1.5 * interquartile range, respectively.

For the local efficiency, indicating the inverse of the shortest path between two ROI, we used the same model structure as for the global efficiency, fitting one model for each of the 358 ROI. For 264 ROI the full-null model comparison was significant (FDR corrected, see Supplementary Material). All the ROI in the fronto-parietal-temporal subnetwork found for the local clustering coefficient were also significant for the local efficiency. Equivalently to the analysis for the local clustering coefficient subnetwork, we investigated the effect of degradation intensity by conducting post-hoc full-null model comparisons for each ROI and condition separately, with "cutoff" as fixed and "participant" as random effect in the full model. FDR-corrected results revealed that local efficiency increased with higher degradation across various ROI in both hemispheres for all conditions but predominantly for MS and ST (see Fig. 6). A decrease with higher degradation was obtained only for a few ROI (and not for ST) (see Supplementary Material).

To see which ROI could be associated with processing of melodies, sentences, spectral or temporal acoustic information, we conducted post-hoc likelihood-ratio tests at each cutoff. Overall, we did not find a clearly localisable pattern (see Supplementary Material).

## Discussion

We investigated whether task goals mediated by top-down attentional effects (i.e., attending to melodies or sentences of sung speech stimuli), complement bottom-up acoustic effects in contributing to hemispheric lateralisation for song or speech perception. To do so, we applied graph theoretical metrics on fMRI connectivity matrices extracted when participants attended to melodies or sentences of sung speech stimuli degraded in spectral or temporal dimensions. We had two alternative hypotheses: (1) that attention to melodies or sentences of sung speech would interact with spectral or temporal acoustic cues to contribute to hemispheric lateralisation (interaction hypothesis), or (2) that hemispheric lateralisation depends solely on bottom-up acoustic information, that is spectral or temporal acoustic cues (acoustic hypothesis). Regarding our predictions, the results in our planned analyses partially supported the interaction hypothesis, in particular at the subnetwork level.

First, using the behavioural data from a subset of participants of Ref.^16^, we confirmed that perception of melodic content is most affected by degradation in the spectral dimension, while perception of speech content is most affected by degradation of information in the temporal dimension. These results align well with seminal studies^15,18^ on the robustness of speech comprehension to spectral degradation and vice-versa. However, it may be objected that task difficulty might have been different for the recognition of melodies and sentences, since sentences may be recognised based on a single word while melodies may require the entire stimulus to make a judgement. The difference in variability across cutoffs when non-crucial acoustic cues were degraded (between MT and SS), although not significant, may thus indicate different listening strategies. Nevertheless, the previous study by Ref.^16^ (see their Supplementary Fig. S2) showed that the difficulty of the task was comparable in the case of spectral degradation for melodies and temporal degradation for sentences. Indeed, even if sentences may be recognised on the basis of a single different word, melodies can also be judged to be different on the basis of the first different note. Also, under a perspective of inference of signal identity in noise and given that spectrotemporal filtering impacted each word and note for a given stimulus, it is likely that more than one different word or note is required to achieve a similarity judgement with sufficient confidence^62,63^, i.e. uncertainty is reduced with every new element (note or word), as suggested by information theoretic accounts (cf. Ref.^64^). The fact that participants were non-musicians and were therefore likely more experienced at judging speech than melodies under difficult listening conditions may be an alternative explanation.

For fMRI connectivity matrices, global graph theoretical metrics did not indicate clear evidence for lateralised functional segregation either under the form of an interaction of attention to melody or sentence and acoustic cues (interaction hypothesis) or as a function of acoustic cues alone (acoustic hypothesis). We predicted to observe lateralised segregation differences with different task goals and thus attentional focus. We did not observe a clear pattern regarding our hypotheses in global efficiency or global clustering coefficient. Also, the overall modular organisation into a motor, an occipital, and one or two fronto-temporal-parietal modules remained across conditions (see Supplementary Table S7). Changes in module affiliation between cutoffs and conditions did not significantly affect the modularity index Q. Thus, we cannot conclude that segregation in modularity increases with enhanced attentional demands when compared among different listening tasks (rather than compared with resting state as in Ref.^43^). We also did not obtain any significant change in the within-module centrality of any ROI, suggesting that their roles as hubs within their modules stayed similar across conditions. This suggests that differential reconfiguration of functional connectivity might instead be found below the level of modules in local configurations.

Indeed, we found evidence for differential segregation regarding the local clustering coefficient. The local clustering coefficient of five regions comprising left medial prefrontal (l8BM), left hippocampal (lH), right auditory association (rA5, rSTSvp), and right superior parietal (rAIP) regions were modulated across conditions, roughly reflecting the whole-brain modular organisation we observed (except for the occipital module). In contrast to our expectations, we obtained significant ROI only in auditory but not in cingulo-opercular or ventral attention regions in this analysis. Within the obtained subnetwork, all ROI from both hemispheres showed an increase of local clustering with higher degradations for speech with temporal degradation (ST), while an increase for MS occurred for right lateralised auditory regions (rA5 and rSTSvp) only. Thus, we obtained partially lateralised effects for the conditions where the crucial acoustic information was degraded, partially in line with the interaction hypothesis. Additionally, segregation, as indexed by the local clustering coefficient, increased with higher degradation, possibly reflecting higher attentional demands, as predicted. This means that neighbouring nodes of these ROI in the network increased in their closed-triangle motif structure with the ROI of these networks, leading to tight integration with these ROI, supporting functional specialisation for the task at hand^42^.

The left hemispheric regions lH and l8BM showed higher clustering with increased degradation of temporal cues and attention to sentences (ST). lH has previously (without using Ref.^54^ parcellation) been associated with semantic speech processing, but also emotional valence^65–69^. l8BM has previously (without using Ref.^54^ parcellation) been associated with speech perception and production and musical familiarity and expectation^70–76^. Despite associations with both music and speech processing in these areas, the topological reconfiguration we observed regarding clustering occurred with attention to sentences only. However, in Ref.^77^ assignment of Ref.^54^ parcellation to large-scale brain networks, l8BM has been assigned to the semantic language network, which is more in line with our results. Interestingly, this semantic language network also comprises the left hemispheric homologues of our obtained rSTSvp and rAIP. The left AIP is additionally also part of the dorsal attention network, while the right AIP has previously been associated with motor imagery, co-speech gesture and finger movement^78–82^. Although music perception is often associated with motor processes^83^, we found clustering effects in rAIP to be significant for ST but not MS. In contrast, clustering effects for rSTSvp were significant for both MS and ST. Along with rA5 (part of the auditory network in Ref.^84^), rSTSvp has previously been associated with music and speech syntax, perception and prosodic emotion^85–92^. Given that homologue areas in brain hemispheres can show similar, but not identical functions (e.g. Refs.^16,93^), we propose that rAIP and rSTSvp can be considered as homologues to parts of the left-hemispheric semantic language network are related to a judgement of non-semantic meaningfulness, that in speech might be related to prosodic perception and in music to melody perception. In contrast, the left hemispheric l8BM might be related to processing meaningful propositional information. Since the task goal confers meaning to aspects of the stimuli, these regions may change topographically when the task goal, and thus the meaning (semantic or not), is difficult to access due to the degradation of the relevant acoustic cues.

Complementary, we observed an increase in local efficiency with higher degradation in distibuted ROI bilaterally, but again mainly for MS and ST (see Fig. 6), thus, when spectrotemporal cues were crucial to solving the task and with increasing attentional demands. These changes included the ROI of the clustering coefficient subnetwork. If we interpret changes of efficiency resulting from topological reconfiguration in clustering, these results could mean that ROI that reconfigure their clustering act as a relay station from primary sensory areas to a wider functional network, distributing and integrating this information and recruiting attentional resources. These findings at the subnetwork level reflect the behavioural results.

Thus, we did find significant topographical reconfiguration partially supporting the interaction hypothesis at the subnetwork level but not at the global level, nor regarding the role of ROIs as hubs within their modules. It is possible that the different measures we used index not only different granularity levels, but also different processing stages. Previous literature suggests lateralisation based on spectrotemporal acoustical cues^16,22^ (sentences with temporal acoustical cues left, melodies with spectral acoustical cues right). However, Ref.^22^ (see also Refs.^23,94^) also obtained dedicated areas for speech and music beyond acoustic processing but in bilateral secondary auditory areas. The authors suggest that the speech-specific areas reflect early phoneme and syllable processing before the stage of speech intelligibility. Our results suggest that subsequently, when recognising melodies or sentences (thus beyond early processing), differential attentional demands partially recover lateralisation through reconfiguration of motif structure when task-relevant acoustic information is degraded. This reconfiguration influences efficiency in a wide bilateral network. Alternatively, the apparent discrepancy between bilateral or lateralised responses to speech and music could reflect the distinction between the processing of general sound categories, such as speech, music or songs, and the encoding of individual examples of general cognitive dimensions. For instance, while selective responses to auditory categories like voices and music occur bilaterally in associative auditory regions^22,23,94^, processing of individual sentences and melodies occurs in the left and right hemispheres, respectively^16^. In other words, neural response patterns shared across all stimuli of the same domain (voices, music) are present bilaterally, while neural patterns discriminating different instances of the same domain are more focal and lateralised^95^). Finally, the general modular organisation of the entire network and global clustering and efficiency were not significantly changed, and their stability might reflect late task-related processing remaining stable across conditions, such as decision-making for meaningful auditory stimuli.

Our study takes on a particular perspective on the problem of lateralisation for song and speech due to stimulus and task design, which should be complemented in future studies. First, our participants were presented with sung speech stimuli, a common but specific hybrid form of music and language. These stimuli have the advantage of carrying all necessary features to understand melodies and sentences, thus keeping the acoustics constant and changing only the attentional focus. However, they lack features typical for spoken language, especially regarding prosodic features like intonation, the usual variability in syllable frequency and overall tempo, or the pragmatic conversational exchange in turn-taking. Sung music with lyrics is likely perceived as a typical instance of music rather than speech, along with invoking possible expectations and modes of perception (see e.g. Ref.^96^), even if the attentional focus is on sentences. Even when spectral information is filtered out from the stimuli, the resulting percept likely reminds of harsh whispering, which is also no typical mode of spoken conversation, although not uncommon. The specificity of melodic vocalisations as a proxy for musical perception is less severe since sung music with lyrics is cross-culturally familiar from early childhood throughout the lifespan. Moreover, vocalisations likely were a focal trait for evolutionary niche constructive effects^6,97^. Thus, even if sung speech is common, it might not be generalisable to spoken language. We therefore recommend future studies to use effects like the song-speech illusion. However, a possible drawback would be the need to repeat a stimulus to sound like song, which might introduce other unwanted effects qua repetition (see Ref.^98^).

Second, the fact that melodies or sentences need to be recognised, given that the crucial acoustic information is hard or impossible to perceive, is unusual. However, it might be compared to speech- or music-in-noise (see Ref.^99^), and be, in that sense, a specific case of a general problem the auditory system faces. Thus, although the current tasks were quite specific, the lateralised changes in topology with increasing attentional demands might be generalisable, somewhat in line with the findings of Ref.^43^. We recommend that future studies follow this line of research further.

Third, it might be objected that there are differences in how sentences and melodies are memorised, especially concerning the presence of non-crucial acoustic information (i.e., sentences without melodies and melodies without words). Spoken language is likely more abundant in everyday life than sung music for our participants (Western non-musicians), thus, language might be easier to remember for our participants. This is visible in the behavioural data, which, although remaining constant in both cases, are more variable for melodies with temporal degradation than for sentences with spectral degradation. On the other hand, discrete pitch, as in melodies, might enhance memory for sound sequences^100^. Also, even if underlying cognitive processes differ between working memory for sentences and melodies^101,102^, they appear to get integrated to yield comparable behavioural outcomes between different task goals in our study. Future studies should investigate the variation in everyday exposure to speech and song cross-culturally.

In summary, we found partial evidence for lateralisation regarding the interaction hypothesis in functional connectivity and no clear evidence for the acoustic hypothesis. While the overall modular organisation of functional connectivity remained relatively stable and the overall topological configuration in clustering and efficiency did not indicate a clear pattern regarding specific attentional demands, reconfiguration occurred at submodular stages in clustering in a specific bilateral subnetwork when task-relevant acoustic cues were degraded. This subnetwork might act as a relay station from spectrotemporally specialised auditory regions to recruit attentional resources when attentional demands are high, altering the efficiency of information transfer to remote brain regions as a function of task goal and available acoustical cues. We suggest that lateralisation is reduced from early to later processing stages on behalf of more distributed processing but recovered under higher attentional demands when task-relevant acoustic cues are hard to obtain.

## Materials and methods

The data used for the present study were the same as in Ref.^16^. Please refer to their Supplementary Material for further details.

### Stimuli

Stimuli were based on ten professionally composed melodies with identical rhythms and consisting of 10 tones each. These were crossed with 10 French sentences, consisting of 10 syllables each, resulting in 100 different stimuli (duration of around 4.5 s). These were recorded by a professional, bilingual (English, French) singer at McGill University, Montréal, Canada (48 kHz sampling rate, 24-bit depth) and ramped. Stimuli were degraded in spectral or temporal acoustic components in the modulation power spectrum domain using a method by Ref.^46^. The modulation power spectrum represents the energy modulation across the spectral and temporal dimensions and is created by applying a 2D Fast Fourier Transform on the autocorrelation matrix of the spectrogram of a sound. Degradation in the modulation power spectrum domain reduces the spectrogram's resolution across spectral or temporal dimensions above a certain cut off-value. Via an iterative procedure (see Ref.^46^, the waveform can then be recreated, resulting in a sound deprived of spectral or temporal acoustic information. Based on pilot testing, we used five cut-offs for each spectral (0.6, 1.5, 1.8, 2, 3 cyc/kHz) and temporal (1, 1.5, 2, 2.5, 3.5 Hz) modulations. These represented a wide range of degradation, from almost none to severe. The resulting stimuli were root-mean-square normalised in amplitude.

### Participants

15 French native-speaking participants (8 Females, mean age = 22.2 ± 1.2 years, education 17.33 ± 1.11 years) without musical training participated in the fMRI and behavioural experiment. All participants were right-handed. Participants gave their written informed consent and received monetary compensation for their participation. The study was approved ﻿by and all experiments were carried out in accordance with guidelines approved by the Ethics Committee on Human Research of McGill University (MUCH-REB—2017-332).

### Procedure

#### Behavioural experiment

Participants were seated in a sound-attenuated booth. Stimuli were presented with Sennheiser HD 280 pro headphones at ~ 60 dB SPL, using Presentation Software (Neurobehavioral Systems, Berkeley, CA, USA). The same software was used to record participants' responses. The behavioural experiment consisted of a same-different task in which participants had to judge whether pairs of stimuli, separated by a silent gap of 1 s, were the same or different in terms of the sentence or melody they consisted of (see Fig. 2A). Sentences or melodies were always different in the non-target domain and similar in the target domain for half of the trials. After each stimulus pair, a visual cue appeared, indicating whether the judgement was to refer to the sentence or the melody. Participants could decide between six possible responses: "Same", "Maybe Same", "Same not sure", "Different not sure", "Maybe Different", "Different". These responses were collapsed to "same" or "different" for the analysis. There was no time limit for participants to respond, and no feedback was given about the correctness of their responses. The experiment consisted of 6 blocks. The first was a practice block with ten trials, using familiar acapella songs derived from the internet that were not part of the stimulus pool. Four blocks of 50 trials each followed this block. Stimulus pairs were presented in pseudo-random order. Each possible combination of factors (same/different, attention to sentence/melody, temporal/spectral degradation) was distributed uniformly across the four blocks, and each of the five degradation cutoffs of both temporal and spectral degradations occurred 20 times. All 100 sung speech stimuli were presented twice as the first song in the stimulus pair but with a different type of degradation at each presentation. The last block consisted of 40 trials of non-degraded (original) stimuli, which were used to derive a baseline measure to normalise each participant's behavioural performance. The total duration of the experiment was approximately 90 min.

### Analysis of behavioural data

To measure performance deviation for degraded stimuli from baseline comparable between participants, we computed a normalised score by (raw score − chance)/(baseline score − chance) − 1. The baseline score corresponded to performance with the non-degraded (original) stimuli. Chance level corresponded to 50% correct performance. Thus, a normalised score of 0 indicated no change in performance compared to non-degraded stimuli, while positive and negative values represented an increase or decrease in accuracy, respectively. We then modelled individual data with a linear function to obtain one integrated value per participant, degradation type (spectral/temporal) and attentional focus (sentence/melody). We used these values as a response variable in a simple linear model with cutoff (1 to 5) and task (sentences or melodies) as predictors, using the anova function of the R package "car" (version 3.0–2, Ref.^59^). Collinearity was tested using the function "vif" from the same package.

### fMRI task

Participants' blood-oxygen level (BOLD) was recorded while they listened to 110 blocks of five degraded stimuli (either in the spectral or temporal domain; see Figs. 1 and 3A). A visual cue at the beginning of each block (~ 1 s duration) instructed participants to either attend to the melody or sentence aspect of the respective stimulus. Participants were asked to detect two catch trials where a sentence or melody was repeated (1-back task) to control for attention. All other sentences/melodies were not repeated in each block. Catch trial stimuli had low temporal (3.5 Hz) and low spectral (3 Cyc/kHz) degradation. All participants detected the catch trials. We again used Presentation Software (Neurobehavioral Systems, Albany, CA, USA) to present the stimuli and record participants' responses. Stimuli were presented via Sensimetrics MRI-compatible insert earphones at 70 dB SPL. The experiment consisted of two runs (about 20 min duration per run), and each degradation cutoff of both temporal and spectral degradations was presented five times, resulting in 50 blocks, complemented by four blocks of silence and one block of white noise (thus, 55 blocks per run). Between the runs, there was a break of 2 to 3 min. Degradation type (temporal/spectral) and respective cutoffs were distributed equally across each run. The order of presentation was pseudo-randomised, and the same degradation type could be presented a maximum of three times in a row.

### fMRI acquisition parameters and pre-processing

We acquired high-resolution MPRAGE T1-weighted three-dimensional anatomical images using a gradient-echo sequence [192 sagittal slices; time to repetition (TR) = 2300 ms; time to echo (TE) = 2.98 ms; flip angle = 9°; matrix size = 256 × 256; field of view = 256 × 256 mm^2^; voxel size = 1 × 1 × 1 mm^3^]. To measure the whole-brain BOLD signal, we used a gradient-echo EPI pulse sequence (48 axial slices with interleaved, descending, multi-band acquisition (acceleration factor 6); TR, 570 ms; volume acquisition, TE, 300 ms; FA, 50°; 2.5 mm slice thickness; no gap; matrix size, 84 × 84, FOV 210 × 210mm2; voxel size, 2.5 × 2.5 × 2.5 mm^3^): All image pre-processing was performed using the Conn toolbox (version 19c, Ref.^103^) in MATLAB (2017a) with the standard preprocessing pipeline (see https://web.conn-toolbox.org/fmri-methods/preprocessing-pipeline): coregistration and resampling to the first image of the first session (b-spline interpolation), slice-timing correction, outlier identification, normalisation into standard MNI space and segmentation into grey matter, white matter and CSF tissue, and functional smoothing (spatial convolution with a Gaussian kernel of 8 mm full-width half maximum). Denoising was done using Conn's standard pipeline (linear regression of potential confounding effects in the BOLD signal and temporal band-pass filtering; see https://web.conn-toolbox.org/fmri-methods/denoising-pipeline).

### ROI-to-ROI analysis and network generation

**Figure 7:**
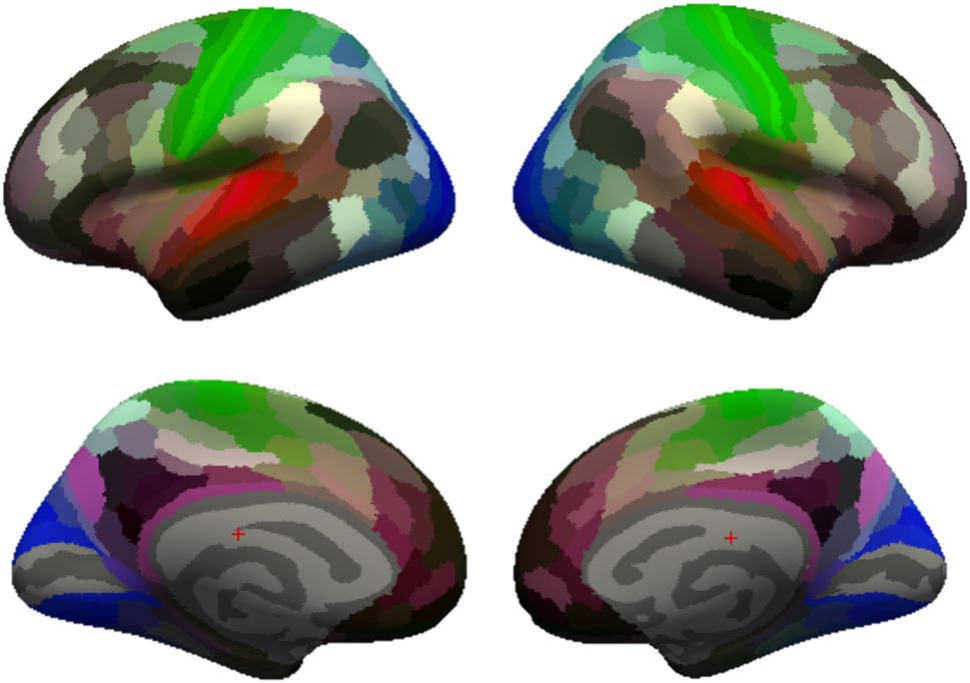
Illustration of the 179 ROI per hemisphere resulting from the conjugation with the Atlas by Ref.^54^. Annotation file created by Ref.^104^.

Functional connectivity analysis was performed using the CONN-fMRI toolbox for SPM (http://www.nitrc.org/projects/conn). Temporal correlations were computed between the BOLD signals of 358 ROI from Ref.^54^ (see Fig. 7). To do so, a general linear model was fitted to analyse the BOLD activity of each participant for each condition. Data were band-pass filtered (0.008–0.09 Hz), and nuisance covariates were included to control for fluctuations in BOLD signal resulting from cerebrospinal fluid, white matter, and their derivatives. It resulted in ROI-to-ROI correlation matrices representing the level of functional connectivity between each pair of ROIs. Each element in the ROI-to-ROI correlation matrix is defined as the Fisher-transformed bivariate correlation coefficient between a pair of ROI BOLD timeseries. This analysis was performed as a function of attention (sentences/melodies), degradation type (spectral/temporal) and cutoff value (5 steps per degradation type) for each of the 15 participants. This resulted in 300 358 × 358 functional connectivity matrices (20 per participant, see Table 3).

**Table 3:** Conditions investigated in our analyses.

Attention	Degradation	
	Spectral	Temporal
Melody	*MS*	MT
Sentence	SS	*ST*

Each condition has five degradation steps. MS and ST are the conditions where crucial acoustical information is missing.

### Graph theoretical analyses

We applied several analyses using measures from Graph Theory (GraphVar toolbox version 2.0, Refs.^52,53^), a field investigating networks regarding their topological organisation. It models brain connectivity networks as "nodes" (ROI) connected by "links" (or "edges"), which in our case were the Fisher z-transformed correlations between ROI. Graph measures can be calculated regarding the entire functional connectivity network (global measures) and subnetworks or single nodes (local measures, see Ref.^42^).

### Global graph measures

Using the GraphVar toolbox, we derived two global measures, the global clustering coefficient and global efficiency, to see whether there are connectivity differences across the entire network between conditions according to our hypotheses. The global clustering coefficient measures, across all nodes, whether the neighbours of a given node are also connected to each other, making it robust against perturbations and providing an indirect measure of functional specialisation if specialisation utilises tight integration in closed triangle motifs (see Ref.^42^). Global efficiency, in contrast, measures the efficiency of information flow via the shortest paths across the network and provides a measure of integration. We used only positive correlations ("negative weights to zero"), a common approach to avoid biasing of graph measures by polarity. Since graph measures depend on the number of connections in the analysis, we used relative network thresholds across a broad range, retaining the strongest 10%, 20%, 30%, 40% or 50% of the edges (cells of the ROI-to-ROI connectivity matrices) ("relative thresholds" 0.1, 0.2, 0.3, 0.4, 0.5). We also created random networks preserving the degree, weight, and strength distribution of the original networks ("c_null_model_und_sign") to normalise our graph measures (100 per participant with ten iterations each). We calculated graph measures for weighted, undirected networks.

### Statistical Analysis of clustering coefficient and efficiency

According to our predictions, we aimed to quantify whether the interaction of attention to melody or sentences with spectral or temporal degradation steps influenced the topological connectivity across the entire network. First, we integrated the derived measures across the different network thresholds (area under the curve, Refs.^55,56^) to reduce multiple comparisons and increase model stability. All statistical analyses were done in R^57^. We fitted the models for local and global efficiency and clustering coefficient using the function "lmer" (R package "lme4"; version 1.1–21; Ref.^60^. We used GLMMs including the fixed factors "attention" (speech or melody), "degradation" (spectral or temporal degradation), "cutoff" (5 cutoffs from high to low degradation) and their interactions, as well as "participants" as random effect and random slopes of all fixed effects within "participants".

We assessed collinearity by fitting a linear model without random effects and interactions between fixed effects and applying the function "vif" (R package "car"; version 3.0–2; Ref.^59^). We assessed model stability using a custom-built function to assess how robust the model estimates would be given changes in the predictors. This function excludes random effects one at a time from the data. Model estimates of the reduced and the full datasets are then compared (please see Ref.^105^ for a comparable approach).

To test the effect of all predictors as a whole, we compared the full model with a null model lacking the predictors of interest, i.e., including only the random intercept and slopes. Models were compared using a likelihood ratio test, applying the function "anova" (argument test set to "Chisq"; Refs.^106,107^. Effect sizes were calculated using the function r.squaredGLMM (R package "MuMIn"; version 1.47.1; Ref.^108^).

### Modularity analysis

To investigate modularity using GraphVar, we again used only positive weights ("negative weights to zero") and normalised the matrices with 100 random networks per subject ("null_model_und_sign", ten iterations). We used a Louvain algorithm with 1000 iterations and a resolution (gamma) of 1.0, first at the individual participant level and then at the group level. The algorithm reaches a consensus classification by executing a modular decomposition on a co-classification matrix, which itself is based on iterative classifications of nodes (see GraphVar manual and Ref.^109^ for details). For each condition, participant and cutoff, we calculated an agreement matrix across five relative network thresholds (0.1, 0.2, 0.3, 0.4, 0.5), which became the basis for calculating the modularity metrics, i.e. the modularity index Q and the classification consistency z^110^.

### Supplementary Information

Supplementary Information.

## Data Availability

Raw data, additional tables and plots are available in the OSF repository (https://osf.io/merwk/), code is available from the corresponding authors upon reasonable request.

## References

[1] Fitch WT (2006). The biology and evolution of music: A comparative perspective. Cognition.

[2] Haiduk F, Fitch WT (2022). Understanding design features of music and language: The choric/dialogic distinction. Front. Psychol..

[3] Jarvis ED (2019). Evolution of vocal learning and spoken language. Science.

[4] Kirby S, Tamariz M, Cornish H, Smith K (2015). Compression and communication in the cultural evolution of linguistic structure. Cognition.

[5] Rohrmeier M, Zuidema W, Wiggins GA, Scharff C (2015). Principles of structure building in music, language and animal song. Philos. Trans. R. Soc. B Biol. Sci..

[6] Tomlinson G (2015). A Million Years of Music: The Emergence of Human modernity.

[7] Krumhansl CL (2001). Cognitive Foundations of Musical Pitch.

[8] Ozaki, Y. *et al.* Globally songs are slower, higher, and use more stable pitches than speech [Stage 2 Registered Report]. *Peer Community Regist. Reports* (2023). 10.1126/sciadv.adm9797 PMC11095461 38748798

[9] Elhilali M (2019). Modulation Representations for Speech and Music.

[10] Poeppel D, Assaneo MF (2020). Speech rhythms and their neural foundations. Nat. Rev. Neurosci..

[11] Ding N (2017). Temporal modulations in speech and music. Neurosci. Biobehav. Rev..

[12] Mantell JT, Pfordresher PQ (2013). Vocal imitation of song and speech. Cognition.

[13] Kob M (2011). Analysing and understanding the singing voice: Recent progress and open questions. Curr. Bioinform..

[14] Sundberg J (1989). The Science of the Singing Voice.

[15] Shannon RV, Zeng F, Kamath V, Wygonski J, Ekelid M (1995). Speech recognition with primarily temporal cues. Science.

[16] Albouy P, Benjamin L, Morillon B, Zatorre RJ (2020). Distinct sensitivity to spectrotemporal modulation supports brain asymmetry for speech and melody. Science.

[17] Albouy P, Mehr SA, Hoyer RS, Ginzburg J, Zatorre RJ (2023). Spectro-temporal acoustical markers differentiate speech from song across cultures. bioRxiv.

[18] Flinker A, Doyle WK, Mehta AD, Devinsky O, Poeppel D (2019). Spectrotemporal modulation provides a unifying framework for auditory cortical asymmetries. Nat. Hum. Behav..

[19] Jamison HL, Watkins KE, Bishop DVM, Matthews PM (2006). Hemispheric specialization for processing auditory nonspeech stimuli. Cereb. Cortex.

[20] Schonwiesner M, Rübsamen R, Von Cramon DY (2005). Hemispheric asymmetry for spectral and temporal processing in the human antero-lateral auditory belt cortex. Eur. J. Neurosci..

[21] Zatorre RJ, Belin P (2001). Spectral and temporal processing in human auditory cortex. Cereb. Cortex.

[22] Norman-Haignere S (2015). Distinct cortical pathways for music and speech revealed by hypothesis-free voxel decomposition. Neuron.

[23] Norman-Haignere SV (2022). A neural population selective for song in human auditory cortex. Curr. Biol..

[24] te Rietmolen N, Mercier M, Trébuchon A, Morillon B, Schön D (2023). Speech and music recruit frequency-specific distributed and overlapping cortical networks. bioRxiv.

[25] Deutsch D, Henthorn T, Lapidis R (2011). Illusory transformation from speech to song. J. Acoust. Soc. Am..

[26] van der Burght CL, Goucha T, Friederici AD, Kreitewolf J, Hartwigsen G (2019). Intonation guides sentence processing in the left inferior frontal gyrus. Cortex.

[27] Prete G, Marzoli D, Brancucci A, Tommasi L (2016). Hearing it right: Evidence of hemispheric lateralization in auditory imagery. Hear. Res..

[28] Prete G, Tommasi V, Tommasi L (2020). Right news, good news! The valence hypothesis and hemispheric asymmetries in auditory imagery. Lang. Cogn. Neurosci..

[29] Hymers M (2015). Neural mechanisms underlying song and speech perception can be differentiated using an illusory percept. Neuroimage.

[30] Bendixen A (2014). Predictability effects in auditory scene analysis: A review. Front. Neurosci..

[31] Morillon B, Baillet S (2017). Motor origin of temporal predictions in auditory attention. Proc. Natl. Acad. Sci. U. S. A..

[32] Sankaran AN, Leonard MK, Theunissen F, Chang EF (2023). Encoding of melody in the human auditory cortex. bioRxiv.

[33] Zalesky A, Fornito A, Bullmore ET (2010). Network-based statistic: Identifying differences in brain networks. Neuroimage.

[34] Den Hartigh RJR, Cox RFA, Van Geert PLC, Magnani L, Bertolotti T (2017). Complex versus complicated models of cognition. Springer Handbook of Model-Based Science.

[35] Rinne T (2007). Distributed cortical networks for focused auditory attention and distraction. Neurosci. Lett..

[36] Fritz JB, Elhilali M, David SV, Shamma SA (2007). Auditory attention—Focusing the searchlight on sound. Curr. Opin. Neurobiol..

[37] Zatorre RJ (2022). Hemispheric asymmetries for music and speech: Spectrotemporal modulations and top-down influences. Front. Neurosci..

[38] Angenstein N, Scheich H, Brechmann A (2012). Interaction between bottom-up and top-down effects during the processing of pitch intervals in sequences of spoken and sung syllables. Neuroimage.

[39] Lee AKC, Larson E, Maddox RK, Shinn-Cunningham BG (2014). Using neuroimaging to understand the cortical mechanisms of auditory selective attention. Hear. Res..

[40] Chennu S (2013). Expectation and attention in hierarchical auditory prediction. J. Neurosci..

[41] Watts DJ, Strogatz SH (1998). Strogatz—Small world network nature. Nature.

[42] Fornito, A., Zalesky, A. & Bullmore, E. T. *Fundamentals of Brain Network Analysis*. doi:10.1016/C2012-0-06036-X. (2016).

[43] Alavash M, Tune S, Obleser J (2019). Modular reconfiguration of an auditory control brain network supports adaptive listening behavior. Proc. Natl. Acad. Sci. U. S. Am..

[44] Quante L, Kluger DS, Bürkner PC, Ekman M, Schubotz RI (2018). Graph measures in task-based fMRI: Functional integration during read-out of visual and auditory information. PLoS One.

[45] Mcgettigan C, Scott SK (2012). Cortical asymmetries in speech perception: What’s wrong, what’s right, and what’s left?. Trends Cogn. Sci..

[46] Elliott TM, Theunissen FE (2009). The modulation transfer function for speech intelligibility. PLoS Comput. Biol..

[47] Hoenig JM, Heisey DM (2001). The abuse of power: The pervasive fallacy of power calculations for data analysis. Am. Stat..

[48] Lenth, R. V. Post Hoc Power : Tables and Commentary. *Dep. Stat. Actuar. Sci.* Technical Report No. 378 (2007).

[49] Kumle L, Võ MLH, Draschkow D (2021). Estimating power in (generalized) linear mixed models: An open introduction and tutorial in R. Behav. Res. Methods.

[50] Cheung VKM, Meyer L, Friederici AD, Koelsch S (2018). The right inferior frontal gyrus processes nested non-local dependencies in music. Sci. Rep..

[51] Rutten S, Santoro R, Hervais-Adelman A, Formisano E, Golestani N (2019). Cortical encoding of speech enhances task-relevant acoustic information. Nat. Hum. Behav..

[52] Waller L (2018). GraphVar 2.0: A user-friendly toolbox for machine learning on functional connectivity measures. J. Neurosci. Methods.

[53] Kruschwitz JD, List D, Waller L, Rubinov M, Walter H (2015). GraphVar: A user-friendly toolbox for comprehensive graph analyses of functional brain connectivity. J. Neurosci. Methods.

[54] Glasser MF (2016). A multi-modal parcellation of human cerebral cortex. Nature.

[55] Bassett DS, Meyer-Lindenberg A, Achard S, Duke T, Bullmore E (2006). Adaptive reconfiguration of fractal small-world human brain functional networks. Proc. Natl. Acad. Sci. U. S. A..

[56] Bassett DS, Nelson BG, Mueller BA, Camchong J, Lim KO (2012). Altered resting state complexity in schizophrenia. Neuroimage.

[57] R Core Team. R: A Language and Environment for Statistical Computing. (2019).

[58] Field A (2005). Andy field—Discovering statistics using SPSS. J. Adv. Nurs..

[59] Fox, J. *et al.* Package “car”: Companion to applied regression. (2011).

[60] Bates, D., Maechler, M., Bolker, B. & Walker, S. lme4: Linear mixed-effects models using Eigen and S4. (2015).

[61] Fischl B (2012). FreeSurfer. Neuroimage.

[62] Vasil J, Badcock PB, Constant A, Friston K, Ramstead MJD (2020). A world unto itself: Human communication as active inference. Front. Psychol..

[63] Bhandari P, Demberg V, Kray J (2022). Predictability effects in degraded speech comprehension are reduced as a function of attention. Lang. Cogn..

[64] Shannon CE (1948). A mathematical theory of communication. Bell Syst. Tech. J..

[65] Koelsch S, Fritz T, Cramon DYV, Müller K, Friederici AD (2006). Investigating emotion with music: An fMRI study. Hum. Brain Mapp..

[66] Mueller K (2011). Investigating brain response to music: A comparison of different fMRI acquisition schemes. Neuroimage.

[67] Trost W, Ethofer T, Zentner M, Vuilleumier P (2012). Mapping aesthetic musical emotions in the brain. Cereb. Cortex.

[68] Bartha L (2003). Medial temporal lobe activation during semantic language processing: fMRI findings in healthy left- and right-handers. Cogn. Brain Res..

[69] Rodd JM, Davis MH, Johnsrude IS (2005). The neural mechanisms of speech comprehension: fMRI studies of semantic ambiguity. Cereb. Cortex.

[70] Wallmark Z, Deblieck C, Iacoboni M (2018). Neurophysiological effects of trait empathy in music listening. Front. Behav. Neurosci..

[71] Seger CA (2013). Clinical practice guideline for the treatment of posttraumatic stress disorder (PTSD). J. Cogn. Neurosci..

[72] Geranmayeh F, Wise RJS, Mehta A, Leech R (2014). Overlapping networks engaged during spoken language production and its cognitive control. J. Neurosci..

[73] Rothermich K, Kotz SA (2013). Predictions in speech comprehension: FMRI evidence on the meter-semantic interface. Neuroimage.

[74] Kung SJ, Chen JL, Zatorre RJ, Penhune VB (2013). Interacting cortical and basal ganglia networks underlying finding and tapping to the musical beat. J. Cogn. Neurosci..

[75] McNealy K, Mazziotta JC, Dapretto M (2006). Cracking the language code: Neural mechanisms underlying speech parsing. J. Neurosci..

[76] Foster NEV, Halpern AR, Zatorre RJ (2013). Common parietal activation in musical mental transformations across pitch and time. Neuroimage.

[77] Briggs RG (2018). A connectomic atlas of the human cerebrum-Chapter 18: The connectional anatomy of human brain networks. Oper. Neurosurg..

[78] Holle H, Gunter TC, Rüschemeyer SA, Hennenlotter A, Iacoboni M (2008). Neural correlates of the processing of co-speech gestures. Neuroimage.

[79] Sadato N, Yonekura Y, Waki A, Yamada H, Ishii Y (1997). Role of the supplementary motor area and the right premotor cortex in the coordination of bimanual finger movements. J. Neurosci..

[80] Jonas M (2007). Do simple intransitive finger movements consistently activate frontoparietal mirror neuron areas in humans?. Neuroimage.

[81] Péran P (2010). Mental representations of action: The neural correlates of the verbal and motor components. Brain Res..

[82] Garbarini F (2014). Drawing lines while imagining circles: Neural basis of the bimanual coupling effect during motor execution and motor imagery. Neuroimage.

[83] Zatorre RJ, Chen JL, Penhune VB (2007). When the brain plays music: Auditory-motor interactions in music perception and production. Nat. Rev. Neurosci..

[84] Baker CM (2018). A connectomic atlas of the human cerebrum-Chapter 4: The medial frontal lobe, anterior cingulate gyrus, and orbitofrontal cortex. Oper. Neurosurg..

[85] Rogalsky C, Rong F, Saberi K, Hickok G (2011). Functional anatomy of language and music perception: Temporal and structural factors investigated using functional magnetic resonance imaging. J. Neurosci..

[86] Sammler D (2013). Co-localizing linguistic and musical syntax with intracranial EEG. Neuroimage.

[87] Angulo-Perkins A (2014). Music listening engages specific cortical regions within the temporal lobes: Differences between musicians and non-musicians. Cortex.

[88] Park M (2015). Sadness is unique: Neural processing of emotions in speech prosody in musicians and non-musicians. Front. Hum. Neurosci..

[89] Kyong JS (2014). Exploring the roles of spectral detail and intonation contour in speech intelligibility: An fMRI study. J. Cogn. Neurosci..

[90] Bianco R (2016). Neural networks for harmonic structure in music perception and action. Neuroimage.

[91] Hesling I, Dilharreguy B, Clément S, Bordessoules M, Allard M (2005). Cerebral mechanisms of prosodic sensory integration using low-frequency bands of connected speech. Hum. Brain Mapp..

[92] Humphries C, Sabri M, Lewis K, Liebenthal E (2014). Hierarchical organization of speech perception in human auditory cortex. Front. Neurosci..

[93] Sammler D, Grosbras MH, Anwander A, Bestelmeyer PEG, Belin P (2015). Dorsal and ventral pathways for prosody. Curr. Biol..

[94] Boebinger D, Norman-Haignere SV, McDermott JH, Kanwisher N (2021). Music-selective neural populations arise without musical training. J. Neurophysiol..

[95] Morillon B, Arnal LH, Belin P (2022). The path of voices in our brain. PLoS Biol..

[96] Weidema JL, Roncaglia-Denissen MP, Honing H (2016). Top-Down modulation on the Perception and categorization of identical pitch contours in speech and music. Front. Psychol..

[97] Nishimura T (2022). Evolutionary loss of complexity in human vocal anatomy as an adaptation for speech. Science.

[98] Tierney AT, Patel AD, Breen M (2018). Acoustic foundations of the speech-to-song illusion. J. Exp. Psychol. Gen..

[99] McDermott JH (2009). The cocktail party problem. Curr. Biol..

[100] Haiduk F, Quigley C, Fitch WT (2020). Song is more memorable than speech prosody: Discrete pitches aid auditory working memory. Front. Psychol..

[101] Schulze K, Koelsch S, Williamson V, Bader R (2018). Auditory working memory. Springer Handbook of Systematic Musicology.

[102] Albouy P (2019). Specialized neural dynamics for verbal and tonal memory: fMRI evidence in congenital amusia. Hum. Brain Mapp..

[103] Whitfield-Gabrieli S, Nieto-Castanon A (2012). Conn: A functional connectivity toolbox for correlated and anticorrelated brain networks. Brain Connect..

[104] Mills, K. HCP-MMP1.0 projected on fsaverage. figshare. Dataset. 10.6084/m9.figshare.3498446.v2. (2016).

[105] Nieuwenhuis R, de Grotenhuis M, Pelzer B (2012). Influence.ME: Tools for detecting influential data in mixed effects models. R. J..

[106] Dobson AJ (2002). An Introduction to Generalized Linear Models.

[107] Forstmeier W, Schielzeth H (2011). Cryptic multiple hypotheses testing in linear models: Overestimated effect sizes and the winner’s curse. Behav. Ecol. Sociobiol..

[108] Barton, K. MuMIn: multi-model inference. http://r-forge.r-project.org/projects/mumin/ (2009).

[109] Blondel VD, Guillaume JL, Lambiotte R, Lefebvre E (2008). Fast unfolding of communities in large networks. J. Stat. Mech. Theory Exp..

[110] Fornito A, Harrison BJ, Zalesky A, Simons JS (2012). Competitive and cooperative dynamics of large-scale brain functional networks supporting recollection. Proc. Natl. Acad. Sci. U. S. A..

