# Supplementary material for: Spectrotemporal cues and attention jointly modulate fMRI network topology for sentence and melody perception
Source: Sci Rep. 2024 Mar 6;14:5501. doi: 10.1038/s41598-024-56139-6 (PMC10917817; doi:10.1038/s41598-024-56139-6)
Supplement: Supplementary file 1 — Supplementary Information. [file 41598_2024_56139_MOESM1_ESM.pdf]

Spectrotemporal Cues and Attention jointly modulate fMRI Network Topology for Sentence and Melody Perception

Felix Haiduk[1,2*], Robert J. Zatorre[3,4],Lucas Benjamin[3,5], Benjamin Morillon[6], Philippe Albouy[3,4,7]

[1] Department of Behavioral and Cognitive Biology, University of Vienna, Vienna, Austria

[2] Department of General Psychology, University of Padua, Padua, Italy

[3] Cognitive Neuroscience Unit, Montreal Neurological Institute, McGill University, Montreal, QC, Canada

[4] International Laboratory for Brain, Music and Sound Research (BRAMS) - CRBLM, Montreal, QC, Canada

[5] Cognitive Neuroimaging Unit, CNRS ERL 9003, INSERM U992, CEA, Université Paris-Saclay, NeuroSpin center, 91191 Gif/Yvette, France;

[6] Aix Marseille University, Inserm, INS, Institut de Neurosciences des Systèmes, Marseille, France

[7] CERVO Brain Research Centre, School of Psychology, Laval University, Quebec, QC, Canada

*felix.haiduk@univie.ac.at

Behavioural data

Table S1: Estimates and confindence intervals for the linear model of the behavioural data

**Fixed effect**	**Estimate**	**Std. Error**	**t value**	**CI lower (2.5 %)**	**CI upper (97.5 %)**
(Intercept)	-0.220	0.029	-7.522	-0.278	-0.162
cutoffcutoff2	0.040	0.041	0.967	-0.041	0.121
cutoffcutoff3	0.107	0.041	2.579	0.025	0.188
cutoffcutoff4	0.160	0.041	3.868	0.079	0.241
cutoffcutoff5	0.247	0.041	5.964	0.165	0.328
attentionSentence	0.213	0.041	5.158	0.132	0.295
filteringtemporal	0.293	0.041	7.092	0.212	0.375
cutoffcutoff2:attentionSentence	-0.060	0.058	-1.026	-0.175	0.055
cutoffcutoff3:attentionSentence	-0.100	0.058	-1.710	-0.215	0.015
cutoffcutoff4:attentionSentence	-0.160	0.058	-2.735	-0.275	-0.045
cutoffcutoff5:attentionSentence	-0.253	0.058	-4.331	-0.368	-0.138
cutoffcutoff2:filteringtemporal	-0.067	0.058	-1.140	-0.182	0.048
cutoffcutoff3:filteringtemporal	-0.147	0.058	-2.507	-0.262	-0.032
cutoffcutoff4:filteringtemporal	-0.153	0.058	-2.621	-0.268	-0.038
cutoffcutoff5:filteringtemporal	-0.327	0.058	-5.585	-0.442	-0.212
attentionSentence:filteringtemporal	-0.713	0.058	-12.195	-0.828	-0.598
cutoffcutoff2:attentionSentence:filteringtemporal	0.240	0.083	2.901	0.077	0.403
cutoffcutoff3:attentionSentence:filteringtemporal	0.347	0.083	4.191	0.184	0.510
cutoffcutoff4:attentionSentence:filteringtemporal	0.413	0.083	4.997	0.250	0.576
cutoffcutoff5:attentionSentence:filteringtemporal	0.673	0.083	8.140	0.510	0.836

Global clustering coefficient

Table S2: Estimates and confindence intervals for the GLMM for the global clustering coefficient (area under the curve)

**Fixed effect**	**Estimate**	**Std. Error**	**t value**	**CI lower (2.5 %)**	**CI upper (97.5 %)**
(Intercept)	0.458	0.018	25.598	0.422	0.491
attentionS	-0.003	0.023	-0.125	-0.046	0.043
degradationT	0.014	0.023	0.600	-0.036	0.059
cutoffcutoff2	-0.015	0.024	-0.624	-0.066	0.033
cutoffcutoff3	0.020	0.023	0.833	-0.024	0.068
cutoffcutoff4	0.023	0.024	0.954	-0.021	0.072
cutoffcutoff5	0.028	0.023	1.205	-0.018	0.070
attentionS:degradationT	-0.038	0.033	-1.154	-0.107	0.029
attentionS:cutoffcutoff2	0.020	0.033	0.608	-0.046	0.082
attentionS:cutoffcutoff3	-0.026	0.033	-0.786	-0.094	0.034
attentionS:cutoffcutoff4	-0.003	0.033	-0.089	-0.064	0.061
attentionS:cutoffcutoff5	-0.007	0.033	-0.224	-0.072	0.058
degradationT:cutoffcutoff2	0.045	0.033	1.380	-0.017	0.110
degradationT:cutoffcutoff3	<0.001	0.033	-0.010	-0.067	0.067
degradationT:cutoffcutoff4	-0.052	0.033	-1.600	-0.110	0.013
degradationT:cutoffcutoff5	-0.083	0.033	-2.539	-0.144	-0.019
attentionS:degradationT:cutoffcutoff2	-0.008	0.046	-0.172	-0.101	0.082
attentionS:degradationT:cutoffcutoff3	0.015	0.046	0.328	-0.085	0.105
attentionS:degradationT:cutoffcutoff4	0.032	0.046	0.687	-0.060	0.119
attentionS:degradationT:cutoffcutoff5	0.135	0.046	2.917	0.044	0.232

Post-hoc tests

To test which cutoffs drove the interaction "attention:degradation", we fitted GLMMs for each cutoff separately and compared the resulting full (fixed effects "attention" by "degradation", random effect "participant", random slopes of fixed effects within "participant") and null models as post-hoc tests.
Attention and degradation interacted significantly for cutoff 2 (χ^2 = 15.447, df = 3, p = .005, R^2m = .078, R^2c = .831) and cutoff 5 (χ^2 = 12.754, df = 3, p = .001, R^2m = .205, R^2c = .311). We conducted pairwise comparisons with FDR correction as post-hoc tests using the R package "emmeans" (version 1.8.4-1; Lenth, 2023). The interaction in cutoff 2 was driven by a significantly lower clustering coefficient of MS (mean = 0.442, SE = 0.016) compared to MT (mean = 0.501, SE = 0.019) (t.ratio = -3.518, df = 17.5, $p_{fdr\ corrected}$ = .015). The interaction in cutoff 5 was driven by a significantly lower clustering coefficient of MT (mean = 0.416, SE = 0.021) compared to MS (mean = 0.485, SE =

0.01) (t.ratio = 3.278, df = 15.0, $p_{fdr\ corrected}$ = .015) and to ST (mean = 0.503, SE = 0.017) (t.ratio = -3.452, df = 23.2, $p_{fdr\ corrected}$ = .013). Overall, thus, we did not obtain a clear pattern across cutoffs (see Figure S1).

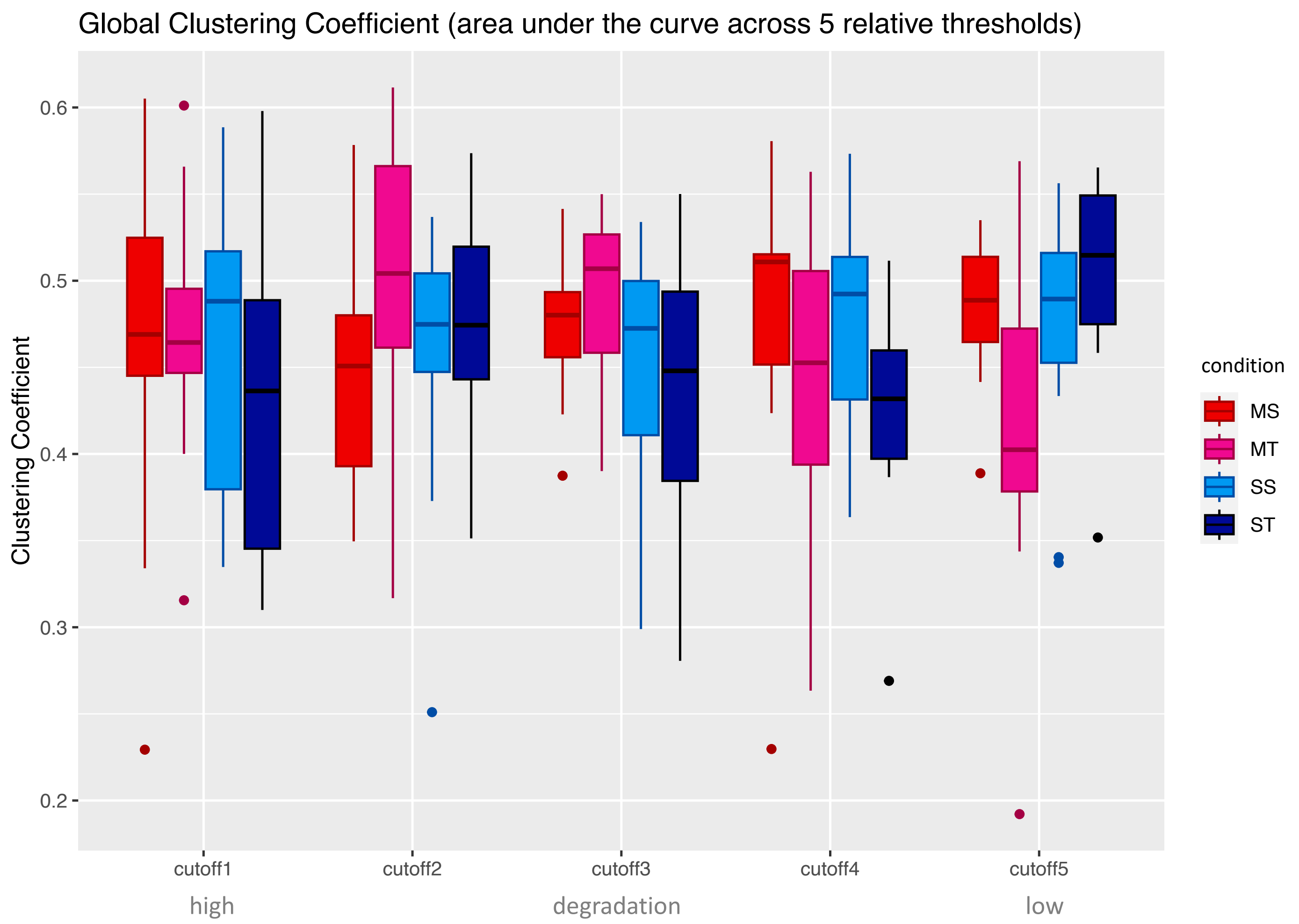

Figure S1: Global clustering coefficient (area under the curve) as function of condition across degradation intensities (cutoff). Boxplot whiskers indicate first quartile - 1.5* interquartile range and third quartile + 1.5 interquartile range, respectively.

<u>Global efficiency</u>

Table S3: Estimates and confindence intervals for the GLMM for the global efficiency (area under the curve)

**Fixed effect**	**Estimate**	**Std. Error**	**t value**	**CI lower (2.5 %)**	**CI upper (97.5 %)**
(Intercept)	0.015	0.002	9.037	0.011	0.018
attentionS	0.003	0.002	1.085	-0.002	0.007
degradationT	-0.002	0.002	-1.020	-0.007	0.002
cutoffcutoff2	0.002	0.002	0.767	-0.003	0.006
cutoffcutoff3	<0.001	0.002	0.006	-0.004	0.005
cutoffcutoff4	-0.002	0.002	-0.691	-0.006	0.003

cutoffcutoff5	0.002	0.002	0.674	-0.003	0.006
attentionS:degradationT	0.001	0.003	0.195	-0.006	0.006
attentionS:cutoffcutoff2	-0.007	0.003	-2.234	-0.013	-0.001
attentionS:cutoffcutoff3	-0.004	0.003	-1.375	-0.011	0.002
attentionS:cutoffcutoff4	0.001	0.003	0.169	-0.006	0.007
attentionS:cutoffcutoff5	-0.001	0.003	-0.350	-0.008	0.005
degradationT:cutoffcutoff2	-0.001	0.003	-0.246	-0.007	0.005
degradationT:cutoffcutoff3	-0.002	0.003	-0.537	-0.008	0.005
degradationT:cutoffcutoff4	0.001	0.003	0.392	-0.005	0.008
degradationT:cutoffcutoff5	0.001	0.003	0.415	-0.005	0.008
attentionS:degradationT:cutoffcutoff2	0.010	0.005	2.226	0.001	0.020
attentionS:degradationT:cutoffcutoff3	0.008	0.005	1.798	<0.001	0.017
attentionS:degradationT:cutoffcutoff4	-0.004	0.005	-0.839	-0.013	0.006
attentionS:degradationT:cutoffcutoff5	-0.004	0.005	-0.824	-0.012	0.005

Post-hoc tests

As for the global clustering coefficient we fitted separate models with the "attention:degradation" interaction as fixed effects (random effect "participant", random slopes of fixed effects within "participant") for each cutoff and ran full-null model comparisons as post-hoc tests.

We obtained significant "attention" by "degradation" interactions for cutoff 2 (χ^2 = 10.961, df = 19, p = .012, R^2m = .16, R^2c = .16) and cutoff 3 (χ^2 = 11.722, df = 19, p = .008, R^2m = .153, R^2c = .327). For cutoff 2, pairwise comparisons revealed that this interaction was driven by a significantly lower efficiency of SS (mean = 0.0118, SE = 0.00158) compared to ST (mean = 0.0195, SE = 0.00199) (t.ratio = -3.020, df = 23.4, $p_{fdr\ corrected}$ = . 036). For cutoff 3, the interaction was driven by a significantly lower efficiency for MT (mean = 0.0106, SE = 0.00124) as compared to ST (mean = 0.0176, SE = 0.00206) (t.ratio = -2.944, df = 21.1, $p_{fdr\ corrected}$ = . 0.046). Overall, thus, we did not obtain a clear pattern across cutoffs (see Figure S2).

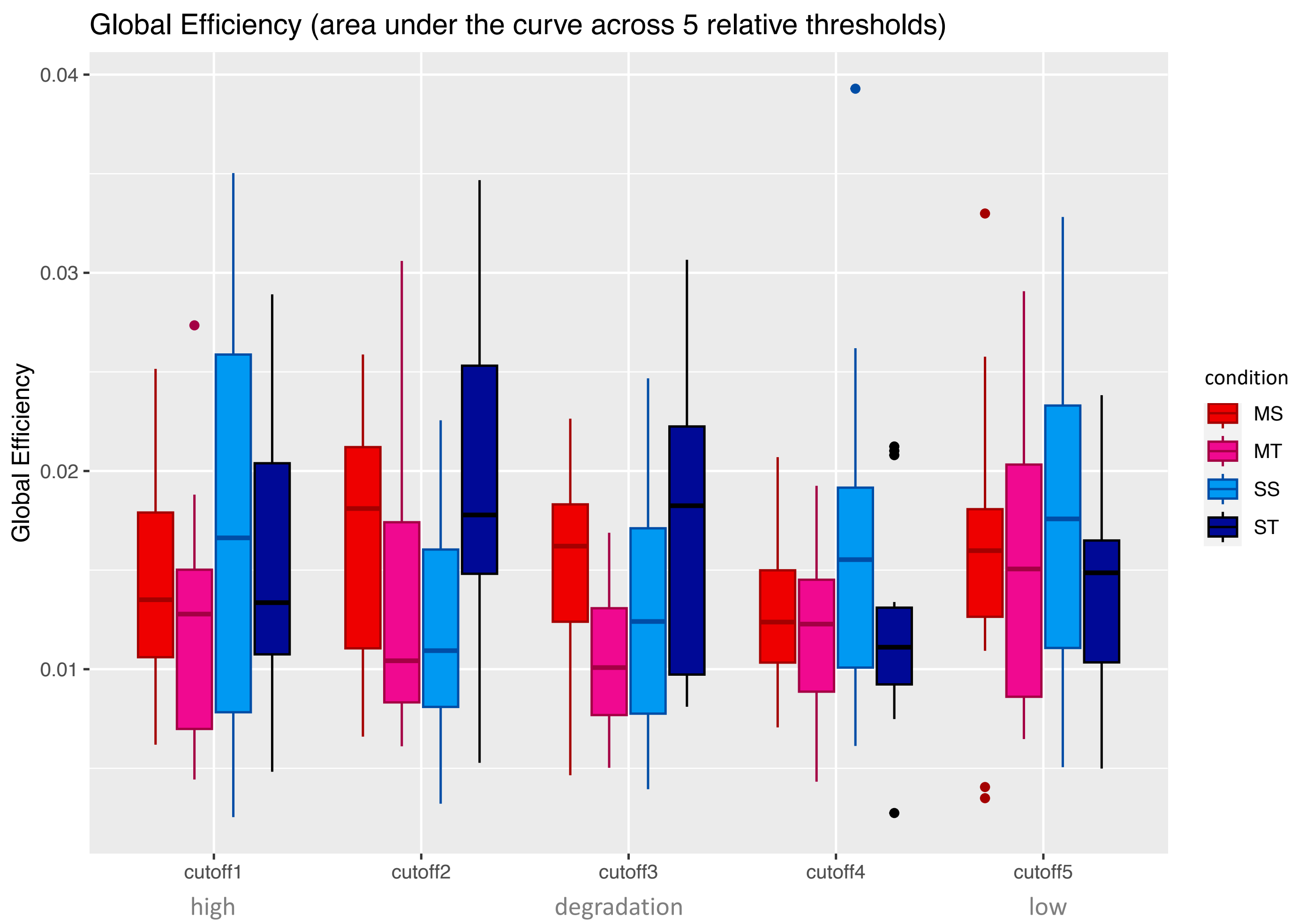

Figure S2: Global efficiency (area under the curve) as function of condition across degradation intensities (cutoff). Boxplot whiskers indicate first quartile - 1.5* interquartile range and third quartile + 1.5 interquartile range, respectively.

Local clustering coefficient

Table S4: Results for the significant full-null model comparisons for the local clustering coefficient.

ROI	χ^2	df	p (FDR corrected)	R^2m	R^2c
l8BM	45.39437	19	0.043	0.127	0.173
lH	45.57780	19	0.043	0.121	0.250
rA5	51.12580	19	0.023	0.121	0.344
rAIP	48.79197	19	0.023	0.119	0.301
rSTSvp	49.00514	19	0.023	0.148	0.158

Table S5: Signifikant likelihood-ratio tests of the interaction of attention and degradation for ROI of the fronto-parietal-temporal subnetwork (local clustering coefficient), by cutoff*

ROI	cutoff	Sum Sq	Mean Sq	NumDF	DenDF	F	Pr(>F)
l8BM	cutoff1	0.001	0.001	1.000	33.953	4.586	0.040

l8BM	cutoff5	0.002	0.002	1.000	38.242	11.853	0.001
l8BM	cutoff4	0.001	0.001	1.000	42.000	4.510	0.040
lH	cutoff1	0.002	0.002	1.000	30.803	6.238	0.018
rA5	cutoff1	0.004	0.004	1.000	26.930	17.213	<0.001
rAIP	cutoff1	0.001	0.001	1.000	33.900	4.193	0.048
rAIP	cutoff2	0.002	0.002	1.000	42.000	8.179	0.007
rSTSvp	cutoff1	0.003	0.003	1.000	56.000	8.767	0.004

*We obtained no significant interaction for lH at cutoff 3, but a main effect of degradation (χ^2=16.215, df=2, p<.001). For the remaining cutoffs of the ROI of the fronto-parietal-temporal subnetwork, no other lower-order effects were significant.

We additionally conducted pairwise comparisons as post-hoc tests (FDR corrected) for each of the ROI at each cutoff to characterise whether they could be specifically associated with processing of sentences or melodies, spectral or temporal processing or their interaction. We did not observe a clear lateralisation according to either of our hypotheses. Instead, regions of the subnetwork were characterised by higher clustering coefficients for specific combinations of attention, degradation and cutoff (see Table S6).

Table S6: Characterisation of ROI of the fronto-parietal-temporal subnetwork (local clustering coefficient) observed. Characterisation was done using post-hoc tests (R package emmeans, version 1.8.4-1) by comparing conditions pairwise (MS,ST,MT,SS). We interpreted conditions with significantly greater estimated marginal means (FDR-corrected) as driving the overall effect for the given ROI. "Ambiguous" refers to ROI that cannot clearly be associated with either condition or factor. Bold: ROI still significant after FDR correction.

**cutoff**	**Spectral Degradation (MS/SS)**	**Crucial degraded (MS/ST)**	**MT**	**Noncrucial degraded (MT/SS)**	**Attention speech (SS/ST)**	**Ambiguous**
1		**rA5**			**rAIP**	l8BM, lH, rSTSvp
2			**rAIP**			
3	**lH**					
4						l8BM
5						**l8BM**

Local efficiency

To see which ROI were differentially related to processing of melodies, sentences, spectral or temporal acoustic information, we conducted post-hoc likelihood-ratio tests at each cutoff. This analysis revealed significant interactions of attention and degradation for 226 ROI, mostly in cutoff 5. 12 ROI showed a main effect of attention, eleven a main effect of degradation (see tables in OSF repository https://osf.io/merwk/). We again characterised ROI according to pairwise post-hoc comparisons. Most ROI had a significantly higher local efficiency for MT and SS (the non-crucial conditions) at cutoff 5 (low degradation). Overall, there was no clearly localisable pattern, but ROI that changed in efficiency for specific interactions of acoustical cues and top-down attention were widely distributed across the brain.

Modularity

To assess whether any ROI would change in their role as core hub within its module as a function of attention, degradation, and cutoff we ran GLMMs for each ROI with using z as a response variable and the fixed factors "attention", "degradation", and "cutoff". We included the three-way interaction of these factors, as well as "participant" as random factor and random slopes of all fixed factors within "participant" (i.e. the same model structure as for Clustering coefficient and Efficiency). The z value indicates classification consistency for each ROI, indicating how often ROIs get co-classified within the same module relative to the other ROI in this module. High consistency values can be interpreted as the node being a core hub of its module, that is as having high within-module centrality [1].
We obtained no significant full-null model comparison for any ROI (FDR corrected), indicating no significant change concerning within-module roles for any ROI.

Exploratorily we also tested whether the condition- and cutoff-specific clustering coefficient of a ROI would predict their within-module functional role as indicated by the z-value, recovering the subnetwork we obtained for the clustering coefficient. We ran GLMMs with the clustering coefficient value, condition and cutoff as fixed effect predictors and participant as random factor (including full random slopes structure). We obtained significant full-null model comparisons and 3-way interactions for 7 ROI (see Table S8). None of those ROI were part to the subnetwork we found for the local clustering coefficient.

Table S7: Module affiliations across subjects and across cutoffs (consensus partition) in left and right for each condition. For the affiliations by cutoff for each condition see OSF repository https://osf.io/merwk/

**Condition**	
**ST**	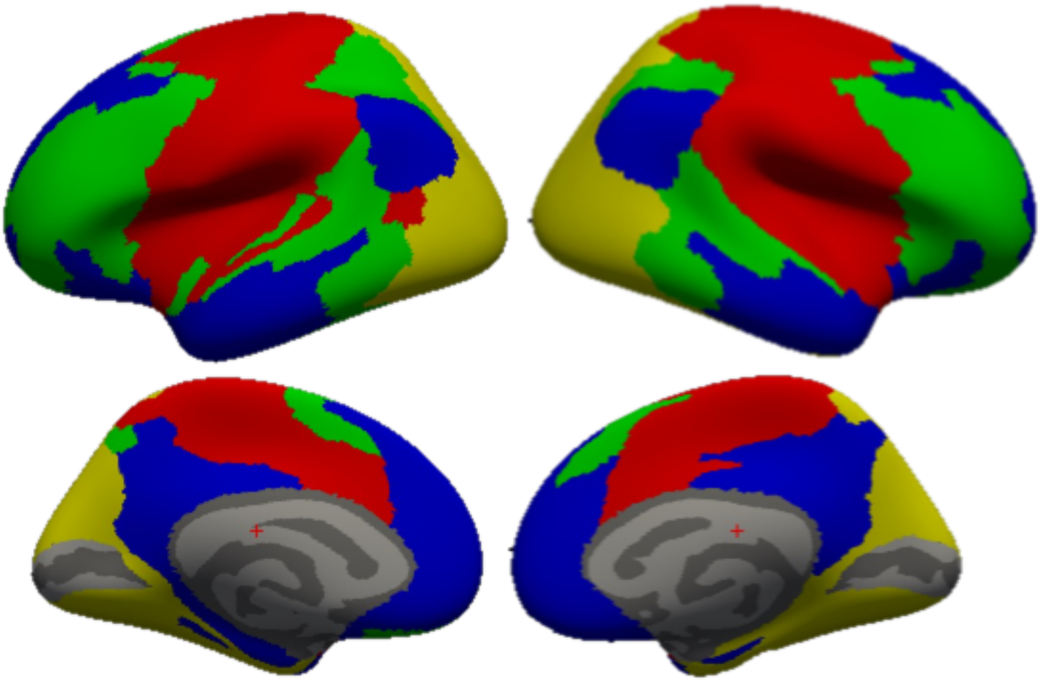
**SS**	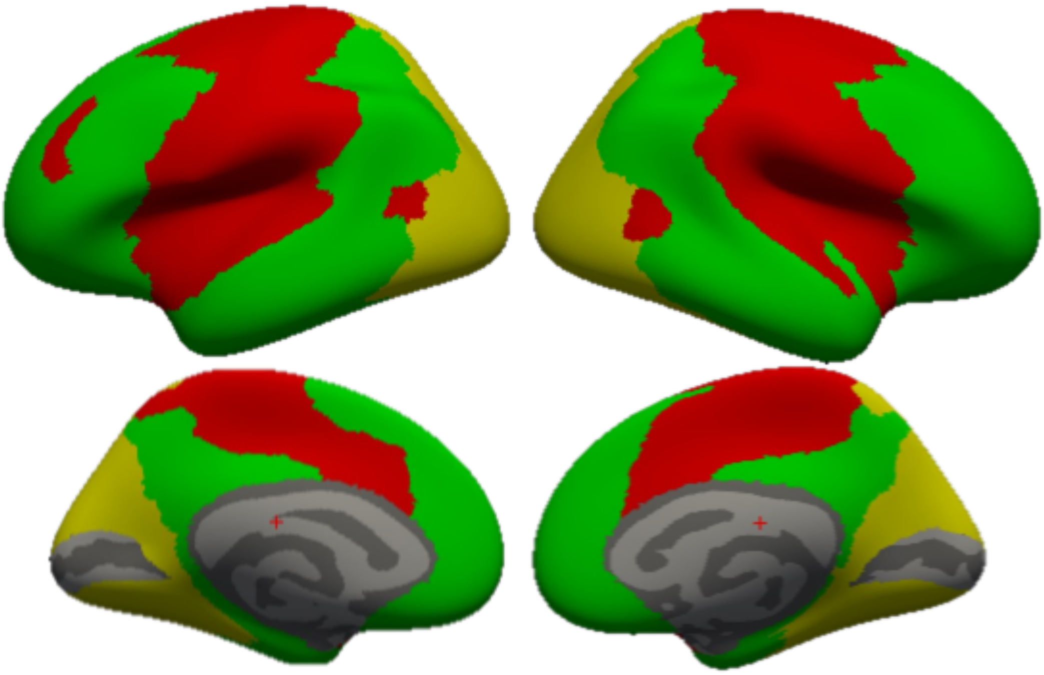

**MT**	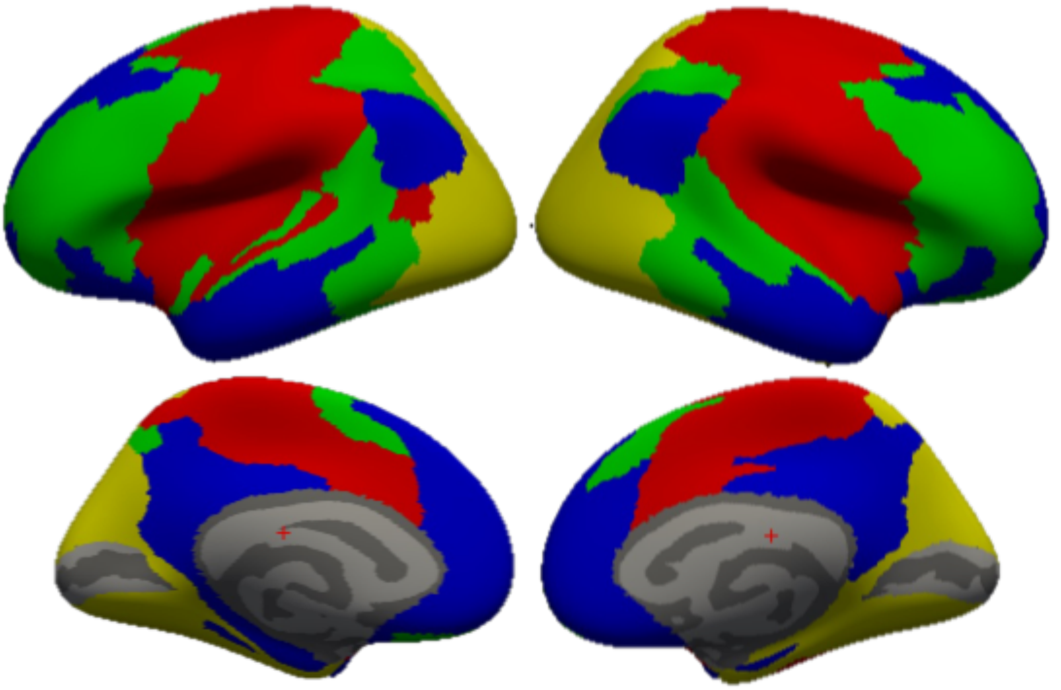
**MS**	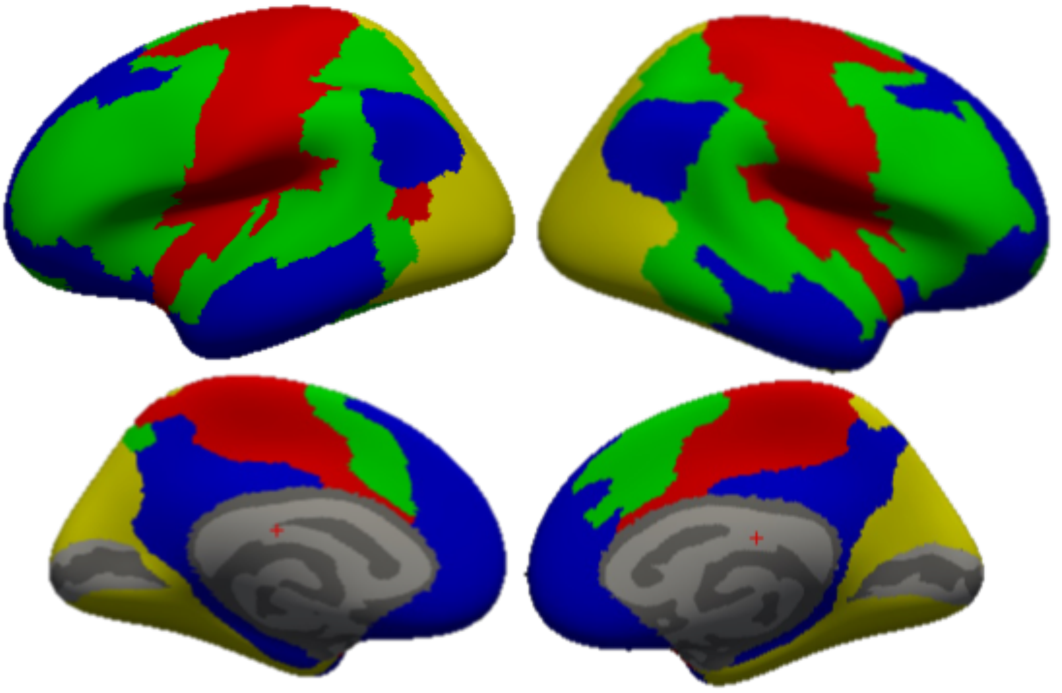

Table S8: ROI for which their clustering coefficient significantly predicted their within-module centrality Z for specific conditions and cutoffs.

**ROI**	χ^2	**Df**	**p (FDR corrected)**	**R^2m**	**R^2c**
lOP2-3	86.112	39	0.004	0.220	0.318
lPBelt	83.960	39	0.005	0.217	0.339
lTPOJ1	75.365	39	0.025	0.198	0.298
rIg	80.065	39	0.011	0.203	0.310
rp32pr	74.472	39	0.027	0.200	0.243
rSTSda	78.122	39	0.014	0.192	0.341

rSTSva	89.217	39	0.003	0.228	0.372

Table S9: Likelihood-ratio tests for the 3-way interaction (z value ~ clustering coefficient * condition * cutoff) for ROI with significant full-null model comparisons.

ROI	Sum Sq	Mean Sq	NumDF	DenDF	F value	Pr(>F)
**lOP2-3**	26.69506	2.225	12	224.926	3.062	>0.001
**lPBelt**	29.073	2.423	12	234.944	2.736	0.002
**lTPOJ1**	28.581	2.382	12	210.650	2.751	0.002
**rIg**	28.986	2.415	12	231.798	3.135	>0.001
**rp32pr**	28.803	2.400	12	223.122	3.147	>0.001
**rSTSda**	24.269	2.022	12	221.135	2.765	0.002
**rSTSva**	22.858	1.905	12	216.315	2.710	0.002

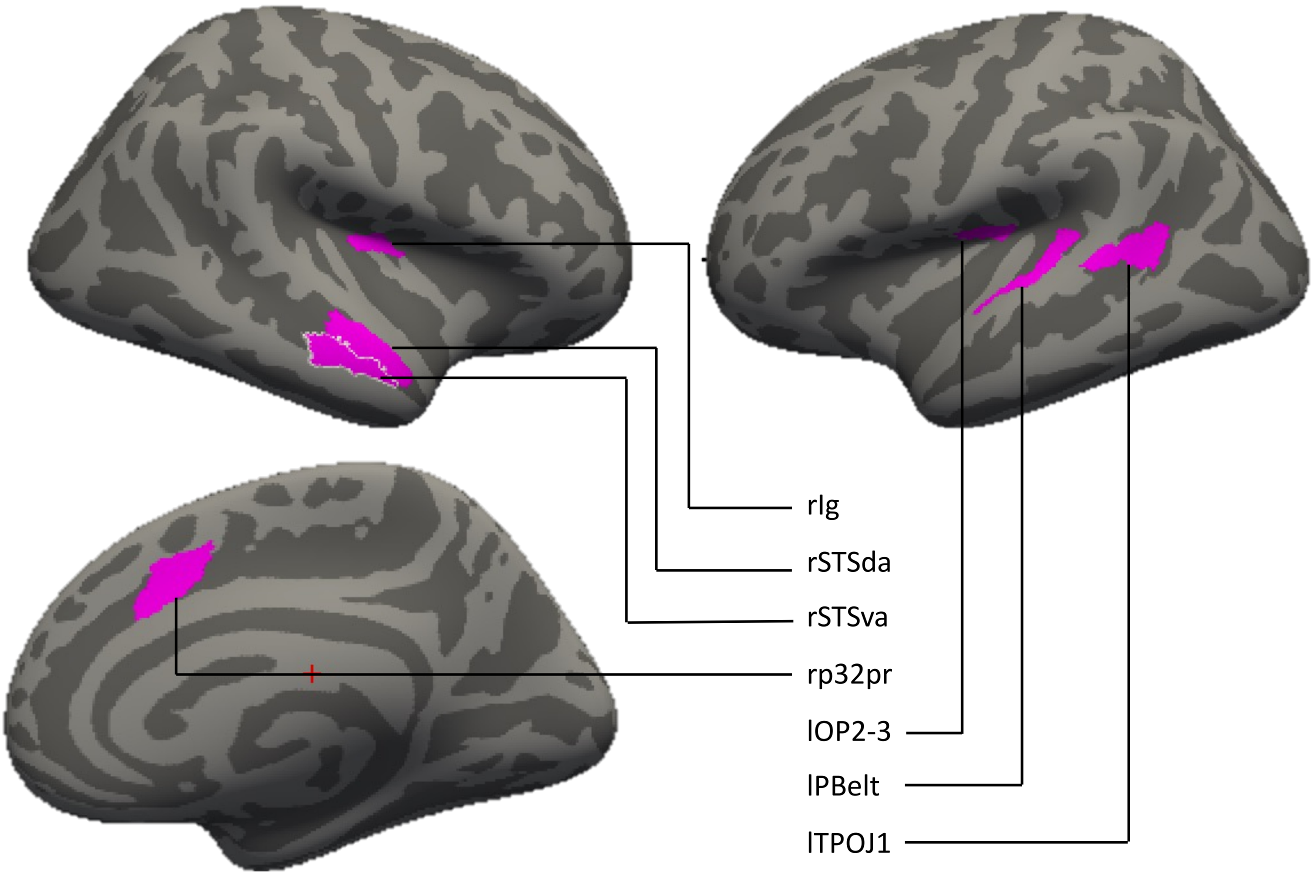

Figure S3: ROI for which their clustering coefficient significantly predicted their within-module centrality Z for specific conditions and cutoffs.

Exploratory Analysis: Network-based statistics

We aimed at identifying whether the participants' behavioural data could be associated with specific subnetworks as a function of condition and cutoff. To this end we used network-based statistics combined with a GLMM framework. The network-based statistic (NBS, [2]) is a mass-univariate approach for identifying statistical effects distributed across subnetworks. That is, the NBS can detect effects that comprise several nodes in a graph (so called components). Recently, the NBS has been combined with the GLMM framework in the R-package "NBS" [3]. We used this package (version 0.1.5) on the subject-level functional 358x358 connectivity matrices to obtain subnetworks associated with participants' behavioural data. We changed the original script "nbr_lme_aov" of the package to accept our GLMM structure comprising "cutoff", "attention", "degradation", and "behaviour" as fixed-effects predictors and "participant" as random effect. We included random slopes of "cutoff", "attention", "degradation" within "participant". The NBS is calculated by running a model for each cell of the upper triangle of the connectivity matrix (i.e. each edge in the graph) and testing the resulting model statistics against a permutation distribution. We used 1000 permutations and calculated the full-null model comparison as well as likelihood ratio tests using reduced models. This would allow us to discriminate resulting NBS components by effects. In order to reduce the computational cost we calculated reduced models only if the full-null model comparison was significant. For the same reason we also only calculated lower-order reduced models only if higher-order models were not significant, since lower-order effects can not be meaningfully interpreted when higher-order effects are present. Since the permutation test is based on the proportion of sampled permutations greater than the observed test statistic, for all models that were not calculated we set the test statistic (χ^2) to 0 and the p-value to 1, rendering them as "non-significant".
We obtained significant NBS components for all effects (higher-order and lower-order), however, these almost always comprised all ROI in the network. We could therefore not interpret the results in a meaningful way regarding our hypotheses (see OSF repository https://osf.io/merwk/ for detailed results and plots).

References

1. Fornito, A., Harrison, B. J., Zalesky, A. & Simons, J. S. Competitive and cooperative dynamics of large-scale brain functional networks supporting recollection. *Proc. Natl. Acad. Sci. U. S. A.* **109,** 12788–12793 (2012).
2. Zalesky, A., Fornito, A. & Bullmore, E. T. Network-based statistic: Identifying differences in brain networks. *Neuroimage* **53,** 1197–1207 (2010).
3. Gracia-Tabuenca, Z. & Alcauter, S. NBR: Network-based R-statistics for (unbalanced) longitudinal samples. *bioRxiv* 2020.11.07.373019 (2020). at <https://www.biorxiv.org/content/10.1101/2020.11.07.373019v1%0Ahttps://www.biorxiv.org/content/10.1101/2020.11.07.373019v1.abstract>
